# Operators and their human–robot interdependencies: implications of distinct job decision latitudes for sustainable work and high performance

**DOI:** 10.3389/frobt.2025.1442319

**Published:** 2025-03-04

**Authors:** Milan R. Wolffgramm, Stephan Corporaal, Aard J. Groen

**Affiliations:** ^1^ Research Group Employability Transition, School of Applied Psychology and Human Resource Management, Saxion University of Applied Sciences, Enschede, Netherlands; ^2^ Centre of Expertise for Technology Education TechYourFuture, Enschede, Netherlands; ^3^ Faculty of Economics and Business, University of Groningen, Groningen, Netherlands

**Keywords:** human–robot interaction, collaborative robot, job decision latitude, work design, mixed method, simulation, innovation, modern sociotechnical systems

## Abstract

The collaborative robot (cobot) has the potential to remove barriers for individual operators when deciding on the deployment of robotics in their work. Ideally, using their opportunities to (re)design work (i.e., job decision latitudes), the operator establishes synergetic human–cobot interdependencies that enable the human–cobot production unit to achieve superior performance and foster more sustainable work perceptions than manual production units. However, it remains scientifically unclear whether the operator is both willing to and capable of using cobot-related job decision latitudes, what this means for designing human–cobot interdependencies, and whether these designs improve unit outcomes. Therefore, we built a manual and three human–cobot production units with distinct job decision latitudes. Forty students participated in the manual production unit and operated one of the human–cobot production units during an assembly simulation. Sophistically accounting for individual differences, the results illustrated that most operators used speed- and task-related job decision latitudes to design their human–cobot interdependencies. These behaviours often led to increased productivity and more motivating working conditions. At the same time, these human–cobot interdependencies frequently resulted in limited human–robot interactions, poor production reliability, and more psychological safety risks. This contribution lays a rich foundation for future research on involving individual operators in developing modern production systems.

## 1 Introduction

The collaborative robot arm (cobot) is gaining popularity in Western manufacturing (International Federation of Robotics, 2023). They are relatively cheap, with highly robust hardware and increasingly intuitive software (Calitz et al., 2017). Unlike traditional industrial robots, the cobot can safely engage in direct and flexible human–robot interdependencies (Weidemann et al., 2023). In line with Clark (1996), one could speak of *interdependence* when the activities of one agent depend on what another agent does (and vice-versa). In such interdependencies, the cobot’s technical capacities (i.e., accuracy, repeatability, and efficiency) are uniquely combined with the operator’s human capacities (i.e., maintenance, troubleshooting, and situational intelligence). By leveraging each other’s capacities, the cobot and operator, as a human–cobot production unit, are likely to outperform manual and fully automated production systems in terms of time, quality, and flexibility and, moreover, create more sustainable work for the operator. Uncovering this likelihood is societally relevant since it directly feeds into recent developments in Western manufacturing and Industry 5.0 policy agendas (Renda et al., 2021; European Commission, 2024; Draghi, 2024) that advocate for more production of small and diverse product series (i.e., high-mix, low-volume production) and good quality of working life (Goujon et al., 2024). However, to achieve this, we must better understand how to constructively involve the individual operator in designing the human–cobot interdependencies. In the following paragraphs, we will elaborate on this approach and emphasize the academic relevance of generating such insights.

Since the operator is the actor closest to the cobot, is most aware of the robotic assistance, and personally needs to achieve better performance and quality of working life, it makes sense to provide the operator with a decisive say on the design of the human–cobot interdependencies. Involving the operator in the design of human–robot interdependencies is, in itself, not groundbreaking. Participatory design research reports on how operators design their social robotics (Gasteiger et al., 2022; Rogers et al., 2022). Moreover, the operator’s role becomes increasingly visible in human–robot design methodology. To illustrate this, Johnson (2014) developed his co-active design method to consider both robot and human capacities when designing human–robot interdependencies. Nonetheless, these contributions fall scientifically short since they capture an engineering bias that leads to static and potentially unsustainable design.

To illustrate, over the last 2 decades, many studies have focused on the role of operators in human–robot interaction design. These studies spanned a wide range of contexts and involved various tasks, such as machine loading (Bauer et al., 2016), drilling (Tian and Paulos, 2021), assembly (Rozo et al., 2013; Schraft et al., 2005), glueing (El Makrini et al., 2018), pick-and-place (Bringes et al., 2013; Çençen, 2019), welding (Laine et al., 2007; Wongphati et al., 2015), and logistics (Unhelkar et al., 2014; Berkers et al., 2023)—extensive literature reviews can be found in Hentout et al. (2019) and El Zaatari et al. (2019). However, except for Schraft et al. (2005), Bringes et al. (2013), and van Dijk et al. (2023), who provided the operator with a few decision-making options, such as deciding on human–robot task allocations, all other interdependencies we encountered were predetermined by engineers and could not be altered by the operator at all.

The most considerable risk of the predetermined design is that the human–robot interdependence works well initially but not in the long run. The operator’s *work perceptions* can change (i.e., internalising all aspects of the tasks and the work environment) (Muschalla et al., 2020). Moreover, work demands can also change (especially in high-mix, low-volume production) (Johansen et al., 2021). Both are problematic. Expensive and scarce engineers must constantly step in to secure the production unit’s performance and sustainability because the predetermined human–robot interdependence does not account for these developments, and the operator cannot adjust it. This is economically unfeasible. The only way to unlock human–cobot interdependence for high-mix, low-volume production is by shifting design-related tasks from the engineer to the operator. Modern sociotechnical systems design theory (MSTS) (de Sitter et al., 1997; Benders et al., 2006; Kuipers et al., 2020; Govers and Van Amelsvoort, 2023) provides evidence-based design principles to organise such operator involvement.

A core design principle in MSTS is to provide the operator with enough *job decision latitude*. Karasek Jr (1979) defined job decision latitude as “the discretion permitted to the worker in deciding how to meet these (work) demands” (p. 285). Using their job decision latitude, the operator can change the human–cobot interdependence before, during, and after running it. This could make human–cobot interdependencies more adaptable and likely to contribute to production units’ *performance* and work perception outcomes—these outcomes are specified in Section 2.4. However, to achieve such outcomes, the operator must constructively use their job decision latitudes, stressing the importance of sufficient *instrumental assistance* for operators unwilling to or incapable of (re)designing their human–cobot interdependencies for the better. Even though job decision latitude has been examined exhaustively since the 1980s, the context where individual operators design their human-cobot interdependencies is very novel and unique. It is, therefore, relevant to study how this concept works in such a context.

Studying whether and how operators would use their job decision latitudes to design their human–cobot interdependencies and what consequences these decisions have for production unit outcomes is innovative and comes with three scientific contributions. First, we adhere to various calls for applying MSTS to new production technologies (Govers and Amelsvoort, 2019; Guest et al., 2022; Parker and Boeing, 2023; Oeij et al., 2023). Second, we help clarify operator-related requirements in contemporary human–robot interactions (Sheridan, 2016; Pratti et al., 2021; Baltrusch et al., 2022; Coronado et al., 2022; Ali et al., 2023). Third, we illustrate various sought-after mechanics, nuances, and conditions that constructively unite technological, organisational, and operator-related factors (Weiss et al., 2021; Parker and Grote, 2022; Govers and van Amelsvoort, 2023; Oeij and Dhondt, 2024).

In pursuit of these scientific contributions, our research goal is to describe in detail how individual operators use their job decision latitude and how this usage implicates the design of the human–cobot interdependencies and the outcomes of human–cobot production units. Explicit attention will be paid to how the outcomes of human–cobot production units differ from those of manual production units. We formulated two descriptive research questions to achieve this research goal:

1. To what extent can and will individual operators use their available job decision latitudes to design human–cobot interdependencies?
2. a. How does using job decision latitude change the design of human–cobot interdependencies? b. Do they achieve better performance and work perception outcomes than a manual work system?

We elaborate further on the concepts under study in Section 2. The mixed method used to measure these concepts in a live simulation is explained in Section 3. The results are described in Section 4. This research endeavour is discussed in Section 5.

## 2 Concepts under study

In this section, we elaborate on the five concepts of our research, namely, job decision latitude (2.1), instrumental assistance (2.2), human–cobot interdependence (2.3), performance (2.4), and work perceptions (2.5). A conceptual overview is provided in Section 2.6.

### 2.1 Job decision latitude

Job decision latitude in human–cobot production units allows operators to (re-)design human–cobot interdependence to meet work demands. The more of these options an operator has, the higher their job decision latitude. Following de Sitter et al. (1997) and Dhondt et al. (2014), we focus on job decision latitude at the individual level. There are four potential job decision latitudes that operators could use to affect the design of prebuilt human–robot interdependence (Wolffgramm et al., 2021). First, the operator can direct the cobot’s movement by pausing and resuming the cobot’s operations. Second, the operator can influence the speed of the cobot’s movement using the cobot’s speed parameter and, in doing so, increase and decrease the pace of the work. Third, the operator can manipulate the cobot’s deployment by changing its task allocation (i.e., the operators allocate their tasks to the cobot or reallocate tasks from the cobot to themselves). Fourth, the operator can modify the cobot’s operation by changing the cobot’s programs (i.e., by deleting or adding new commands). In line with Karasek (1979) and de Sitter et al. (1997), who state that certain design expertise is required to effectively use available job decision latitudes, we must address the issue of instrumental assistance.

### 2.2 Instrumental assistance

Echoing Chou and Robert (2008), we consider instrumental assistance as the provision of “tangible assistance, such as materials and resources necessary for a job and guidance or knowledge needed to complete a task” (p. 210). The importance of instrumental assistance in using technology as intended has been shown frequently (Fenlason and Beehr, 1994). Which instrumental assistance is required depends strongly on the operator’s needs. Venkatesh et al. (2003) stated four relevant facilitating conditions to stimulate user behaviour in their unified theory of acceptance and use of technology (namely, knowledge resources, resources, assistance, and system compatibility). Although these findings are being confirmed in more modern studies (Abbad (2021)), they pay too little attention to what the operator needs while interacting with technology. *Additional instrumental assistance* might be required when the operator is invited to design human–cobot interdependence (e.g., repeating information, providing more information, or offering a helping hand).

### 2.3 Human–cobot interdependence

In line with Johnson (2014), human–cobot interdependence is based on the task division between the operator and the cobot. By deploying the cobot for one or multiple tasks, a shared task execution emerges (e.g., the cobot executes the task, and the operator arranges all prerequisites and monitors the cobot). Consequently, the human–cobot interdependence intensifies when the number of shared tasks increases. Inspired by the levels of the automation literature (Parasuraman et al., 2000), we argue that human–cobot interdependencies can be described in levels. We propose that this so-called ‘human–cobot interdependence level’ is related to the number of shared tasks between the operator and the cobot. To illustrate, in the case of ‘human–cobot interdependence level 3,’ the cobot performs three distinct tasks in collaboration with the operator. At level 0, the cobot is not used, and all tasks are performed by the operator (i.e., manual production). As described in Section 2.1, job decision latitudes could change the height of the interdependence level, but only to a certain extent. Reallocating tasks from or to the cobot would directly impact the interdependence level since the number of shared tasks changes. Other job decision latitudes would instead influence how the cobot executes its tasks (e.g., at a higher or lower operational speed or in a more [in]efficient manner). These adjustments have consequences for the human–cobot interdependence level’s dynamics. The level and dynamics of human–cobot interdependencies will likely affect performance and work perception outcomes.

### 2.4 Performance outcomes

In line with Johnson (2014), who illustrated that human–cobot interdependencies could enhance reliability or efficiency, we use production reliability and productivity to compare the performance outcomes of manual production units with those of human–cobot production units. Following Eaton (2004), we define production reliability as “the probability that a system, component, or part will operate satisfactorily for a specified period of time under specified operating conditions” (p. 1). High production reliability means that many products are produced on time and are of acceptable quality (Huang et al., 2018). Productivity is “the ratio of output to input for a specific production situation” (Rogers, 1998, p. 5). High productivity means it takes less time to complete a product from start to finish (Pilat and Schreyer, 2002). We will also study the operator’s work perceptions to estimate whether these performance outcomes will likely be sustained over time.

### 2.5 Work perception outcomes

Since work perceptions concern both the nature of tasks and the work environment in which they take place, we will study three work perceptions, namely, perceived motivational characteristics, situation awareness, and automation-induced complacency. In this section, we illustrate why these perceptions are likely relevant outcome measures and what they comprise.

To sustain the continuity of its working tasks, applied psychology suggests that the operator’s job must capture sufficient motivational characteristics. Morgeson and Humphrey (2006) compiled a comprehensive list of 12 characteristics, including autonomy, task variety, and job complexity, based on a long research tradition in job design. An overview of these characteristics and their definitions can be found in Supplementary Material S5. The motivational characteristics relate to psychological states and work outcomes such as job satisfaction, internal work motivation, stress, and absenteeism. A lack of perceived motivational characteristics indicates unsustainable work design and raises cause for concern.

Because working closely with advanced technology could result in poor monitoring behaviour and inaccurate responses to system failures [i.e., operator-out-of-the-loop behaviour (Sauer et al., 2016)], two work perceptions related to the work environment are crucial. First, the operator must understand their work system’s status [i.e., situation awareness (Endsley and Kiris, 1995)]. Endsley (1988) introduced three consecutive situation awareness error levels. These were later operationalised by Jones and Endsley (1996). Error level 1 concerns the absence or inaccessibility of important information needed to access a situation. Error level 2 ensues when the operator has all the necessary information but lacks the mental ability to understand the current state of a situation accurately. Error level 3 occurs when the operator understands the current situation but cannot predict its near-future state. If the operator is unaware of the situation, it will likely enact operator-out-of-the-loop behaviour.

Second, the operator must not become overly reliant on the technology they are working with [i.e., automation-induced complacency (Parasuraman et al., 1993)]. Automation-induced complacency occurs when the operator enacts suboptimal monitoring behaviour that could lead to performance decay (Parasuraman and Manzey, 2010). Such behaviour could be provoked when operators work with highly reliable systems or when attention must be divided between an automated and a manual task (Merritt et al., 2019). Automation-induced complacency is highly problematic since it prevents the operator from detecting and resolving automation-related issues on time—in this research, these issues are cobot-related. While such issues could negatively affect the human–cobot production unit’s performance, automation-induced complacency can also pose a health and safety risk to the operator [e.g., human–machine collisions (Merat et al., 2019)]. If the operator blindly follows the cobot, it will likely to result in operator-out-of-the-loop behaviour.

### 2.6 Conceptual overview

**FIGURE 1:**
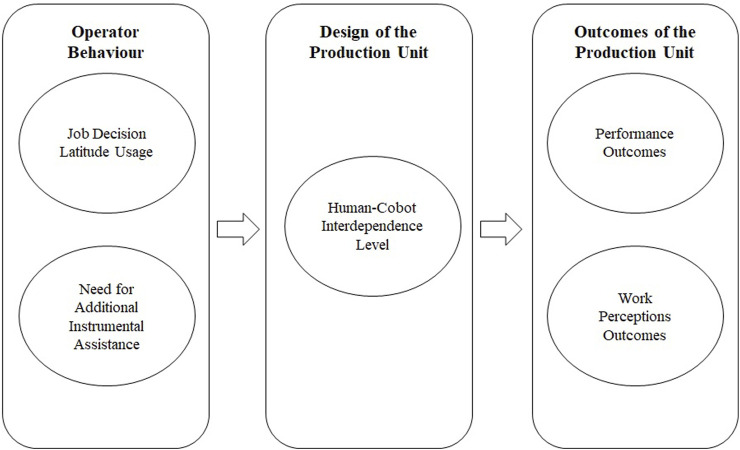
Summary of the framework.

Figure 1 summarises the concepts under study. We want to describe 1) whether and how individual operators use their job decision latitudes to (re)design their human–cobot interdependencies, 2) whether they need additional instrumental assistance, 3) what human–cobot interdependence levels and dynamics these design efforts result in, and 4) whether human–cobot production unit outcomes are superior to those of a manual production unit.

## 3 Method

In the following subsections, we elaborate on the development and analysis of our research. Given the extensiveness of our mixed method, an overview of the data collection and analysis techniques used per sub-concept has been appended (see Supplementary Material S6).

### 3.1 Research setting development

**FIGURE 2:**
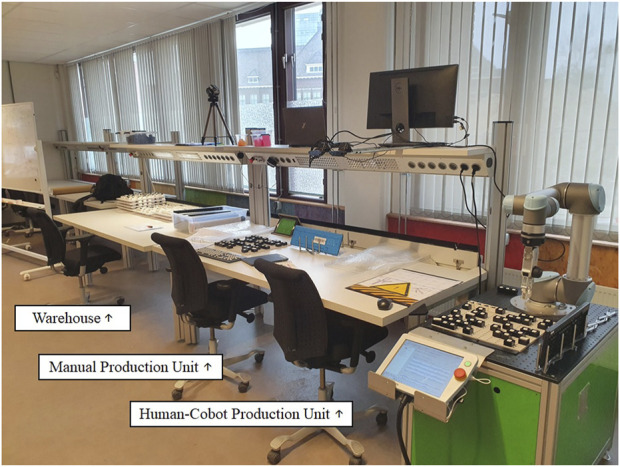
Picture of the detailed design (from left to right: warehouse, manual workstation, and human–cobot production unit).

We developed a research setting in a laboratory environment to carefully research the concepts under study (Falk and Heckman, 2009). First, we determined the setting’s design parameters: multiple job decision latitudes, ample opportunity for different human–cobot interdependence levels, safe and user-friendly cobot applications, features to measure the core concepts and sub-concepts under study, and a manageable procedure. We used the human–robot workstation design method detailed by Ore, et al. (2017) to build our research setting. As per the recommendations of Ore et al. (2017), we planned and clarified the task under study (i.e., assembly), developed the first simple conceptual design (i.e., gateway assembly), and worked toward a more complex embodiment design (i.e., keyboard assembly). The embodiment design was pre-tested with 12 subjects and further developed into a detailed design that captured one warehouse, one manual production unit, and three human–cobot production units with distinct job decision latitudes (Figure 2)—we developed three human–cobot production units since there are no guidelines stating what or how many job decision latitudes would enable the operator to achieve acceptable outcomes. The research setting took 6 months to be operationalised.

### 3.2 Research procedure

**FIGURE 3:**
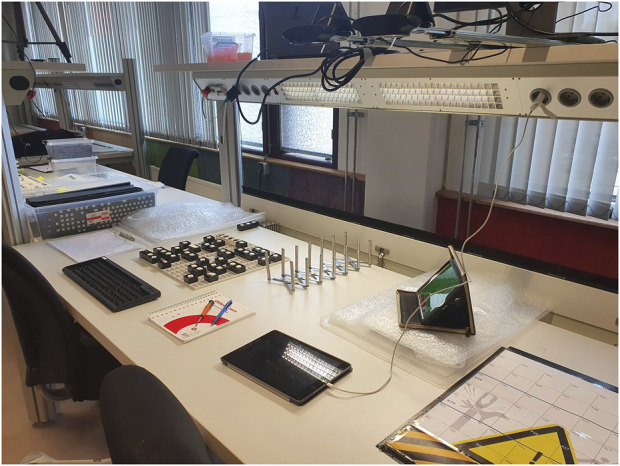
Picture of the manual workstation.

The research procedure was based on a protocolised simulation conducted in Dutch; an English translation of the research protocol can be found in Supplementary Material S1 (the original is available upon request). Individual subjects would fulfil the operator role and be told by a facilitator, who acted as the foreman, that they worked for a manufacturing company that assembled small batches of customised keyboards. The operator would assemble two keyboards of one type and three of another to simulate a high-mix, low-volume production setting. All operators would first assemble the five keyboards at the manual production unit (Figure 3).

**FIGURE 4:**
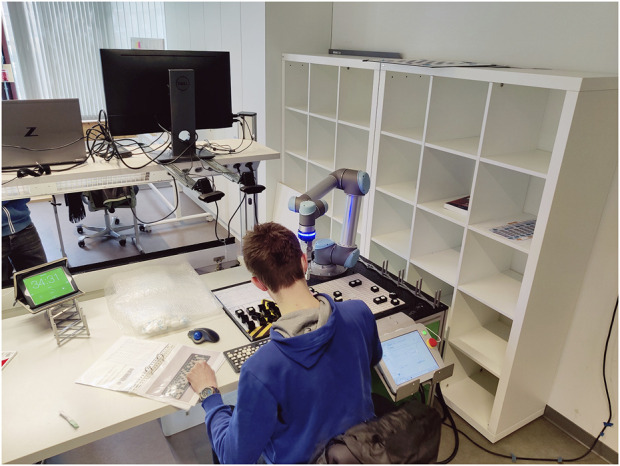
Picture of an operator in the human–cobot production unit.

For each keyboard, identical tasks had to be completed: 1. pick up the empty keyboard, 2. verify it by checking its barcode against the manual, 3. collect the 40 prescribed keys, 4. assemble the keyboard according to the instructions, 5. perform a final check, and 6. hand in the keyboard. The keyboards had to be error-free upon first submission and submitted within seven and a half minutes. Defective keyboards were returned once to the operator for immediate repair. In the second stage, they assembled the same keyboards in one of the three human–cobot production units (Figure 4). The manual session and the work sessions with the cobot would each last 45 min maximally.

The cobot, a Universal Robot (type 5), was equipped with a gripping claw, an LED ring light, a Bluetooth camera, and an external control box. The cobot could execute the following four tasks: 1) getting and handing over keyboards, 2) scanning barcodes with feedback (correct/incorrect), 3) collecting keys and placing these in the right order in front of the operator, and 4) assessing the assembled keyboards with feedback (correct/incorrect). The cobot tasks were captured in separate modules, which the operator had to activate and suppress manually. Moreover, the modules for the two different keyboard types were embedded in different cobot programs. The operator was responsible for switching programs.

By design, each human–cobot production unit had different job decision latitudes (see Table 1 for a schematic overview of the differences per unit). Operators were randomly ascribed to the units based on a card-picking game. In all units, the operator could control the cobot’s motion by temporarily pausing it. In units 2 and 3, the operator could also increase or decrease the cobot’s operational speed and decide on its task allocation, thereby affecting the human–cobot interdependence level, its dynamics, or both—in line with cobot deployment in industrial practice (Bauer et al., 2016), and the human–cobot interdependence level in unit 1 was fixed. Finally, only in unit 3, operators could also change the cobot’s programs by adding or removing commands, altering its actions and performance—changing cobot’s programs could result in more streamlined operations (e.g., fewer halts between modules and more effective operations). Operators were actively encouraged to use their job decision latitudes if they thought these would allow them to make their assembly work more sustainable and achieve better unit performance. The manual work perceptions and performance functioned as a point of reference. Operators had to estimate these manual outcomes by themselves. Feedback was not provided to prevent biased decision-making.

**TABLE 1:** Job decision latitude(s) per human–cobot production unit.

Unit 1	Unit 2	Unit 3
Pausing and resuming the cobot	Pausing and resuming the cobot	Pausing and resuming the cobot
	Changing task allocation	Changing task allocation
	Adjusting the cobot’s speed	Adjusting the cobot’s speed
		Altering cobot programmes

Lastly, considerable instrumental assistance was provided to prevent a lack of working experience with assembly tasks or cobots from affecting our results. Instructions and demonstrations on assembling the keyboards and safely using the cobot were provided to all subjects by the foreman. Specific instructions and demonstrations were provided to operators running human–cobot production units with more job decision latitudes. All workstations had a timer, an assembly manual, a notebook, and a screwdriver to effortlessly remove wrongly inserted keys. Furthermore, a workplace assistant would provide the operator with the keyboards and grids—these plateaus were covered with the 40 transportation sockets that held the keys to be assembled. Moreover, this assistant would answer the operators’ questions and provide expert assistance on request during the simulation, embodying this method’s additional instrumental assistance. All simulations were completed without injury.

### 3.3 Sample

In total, 40 Dutch students participated in this research as operators. Most were enrolled in a technical bachelor’s study (n = 34; 85%) and identified as male (n = 29; 73%); others were enrolled in social studies or attended a technical community college. The sample’s average age was 21.42 (SD 3.05). All operators had limited to no recent working experience with a cobot and received a €15 gift card upon completing the simulation. They were informed about the incentive before participation. Random allocation to the human–cobot production units resulted in the following distribution: 15 in human–cobot production unit 1, 13 in unit 2, and 12 in unit 3. The cycle time of the research procedure, including administering consent, a manual work session, a 15-min break, a collaborative work session, instructions, demonstrations, administering surveys, and a debriefing interview, lasted between 120 and 150 min per operator.

### 3.4 Data collection

All work sessions were video recorded. During the work session, the foreman filled out scorecards about when keyboards were handed in and whether these submissions were on time and error-free upon first submission. After both work sessions, motivational characteristics were measured using a translated version of the validated work design questionnaire (Morgeson and Humphrey, 2006)—the questionnaire was carefully translated by multiple researchers. Alertness questions were added to mitigate the response set (e.g., “I wear blue socks with yellow dots”). Inspired by the Situation Awareness Global Assessment Tool (Endsley and Garland, 2000), three situation awareness assessments, each with different ‘probes,’ were administered during the collaborative work sessions. In line with Parasuraman et al. (1993), a small technical inefficiency was added to one cobot program to assess participants’ automation-induced complacency, commanding the cobot to collect three faulty keys. Finally, semi-structured debriefing interviews were conducted to obtain participants’ opinions about the simulation, their job decision latitude(s), and work perceptions. The translated work design questionnaire, the situation awareness probes, and debriefing questions are presented in Supplementary Materials S2, S3, S4.

### 3.5 Data analysis

We first analysed the data per operator by comparing both its manual and human–cobot production units. These results were later grouped per human–cobot production unit. Multiple researchers analysed video recordings per minute using an observation template (available upon request). The observation outcomes revealed which of the available job decision latitudes were used by the operator, which additional instrumental assistance was required, the human–cobot interdependence levels and dynamics, and whether the complacency checks were handled correctly (i.e., leaving the faulty keys in their transportation sockets). We quantified the observations per keyboard, excluding questions and/or cobot pauses directly related to the complacency checks.

The scorecards were analysed to determine the production reliability (i.e., less or more error-free and timely submissions) and productivity (i.e., longer or shorter handling time) outcomes per keyboard. These outcomes were totalled, averaged, and equipped with a standard deviation if possible. For the motivational characteristics, we checked the work design questionnaires for any response set. Questionnaires with an incorrect manipulation question were excluded from the dataset. In total, 31 manual and 37 collaborative entries were included. The validity of the questionnaire is supported by the convincing work of Morgeson and Humphrey (2006) and Humphrey et al. (2007). Boxplots were created to visualise the developments in productivity and motivational characteristics.

The responses to the situation awareness probes were reviewed and awarded one point per correct answer. Three points could be awarded to each of the three error levels. An error level was considered passed if at least two situation awareness probes were answered correctly. Automation-induced complacency was considered present if the operator took at least one faulty key from its transportation socket during two out of three complacency checks. To better understand the observation and questionnaire outcomes, the debriefing interviews were simultaneously coded by multiple researchers using open codes. The coded quotations functioned as potential explanations or illustrations for the operators’ design behaviour and perceptions (e.g., operators who did not use all job decision latitudes due to a sense of time pressure).

Finally, the human–cobot production unit’s performance was considered better than the manual unit’s if at least three productivity and/or reliability improvements were realised across the five keyboards (e.g., two keyboards with productivity improvements and one keyboard with a reliability improvement). Work perceptions were considered better if a) at least six of the operator’s motivational characteristics increased and b) the operator passed at least two out of three situation awareness error levels or handled two out of three complacency checks successfully. We checked which operators achieved better (or worse) performance and work perception outcomes for each of the three human–cobot production units. The core findings per unit were used to create a summarised overview.

## 4 Results

In this section, we present the results for the manual and each of the three human–cobot production units. At the end of this section, we provide an overview of the key insights per human–cobot production unit.

### 4.1 Manual production unit outcomes

Since most of the performance and work perception outcomes generated by the human–cobot production units will be compared to those generated in manual production, we present the manual outcomes in Table 2. We clustered the individual operators’ manual outcomes and scores based on the human–cobot production unit they were later assigned to (i.e., unit 1, unit 2, or unit 3). Although the manual outcomes, on average, are very similar across operators, we must be aware that for each individual operator, the outcomes generated by the human–cobot production unit are compared to its respective manual outcomes. This means that the height of the operator’s manual outcomes strongly determines the likelihood of being improved by those of the human–cobot production unit. While low manual outcomes are more likely to be surpassed, excellent outcomes and scores cannot be, leading to high heteroscedasticity. This is why we must focus on studying the results at the individual level rather than at the aggregate level as we anticipate substantial individual differences within units in this results section.

**TABLE 2:** Overview of production reliability and productivity in operators’ manual production per keyboard and manual motivational characteristics scores, stratified per production unit.

	Manual production unit 1 (n = 15)			Manual production unit 2 (n = 13)			Manual production unit 3 (n = 12)		
Product	Productivity^a^ (in seconds)		Production reliability^b^ (in products)	Productivity (in seconds)		Production reliability (in products)	Productivity (in seconds)		Production reliability (in products)
	Mean	Standard deviation	Proportion	Mean	Standard devitaion	Proportion	Mean	Standard deviation	Proportion
Keyboard 1	449	75	5/15	416	85	9/13	429	55	7/12
Keyboard 2	373	87	10/15	374	87	8/13	348	54	10/12
Keyboard 3	455	101	9/15	469	148	8/13	447	120	6/12
Keyboard 4	340	58	10/15	343	75	9/13	367	89	10/12
Keyboard 5	327	87	12/15	311	45	12/13	305*	93*	10/11*

Motivational characteristic^c^	Mean**	Standard deviation**	Mean***	Standard deviation***	Mean****	Standard deviation****
Work scheduling autonomy	3.70	0.88	3.63	0.82	3.73	0.72
Decision-making autonomy	3.00	1.21	3.07	0.97	2.97	1.21
Work method autonomy	4.12	1.20	4.23	0.80	3.80	0.80
Task variety	2.03	0.84	2.40	0.86	1.73	0.58
Task significance	2.48	1.10	2.45	1.07	1.95	0.51
Task identity	3.77	0.83	3.80	0.94	3.73	0.77
Feedback from job	3.06	0.70	2.93	1.03	2.90	0.85
Job complexity	1.57	0.43	1.95	0.67	1.48	0.34
Information processing	2.84	0.78	2.75	0.98	2.60	0.71
Problem-solving	1.75	0.79	2.05	0.76	1.90	0.62
Skill variety	2.57	0.87	2.53	0.74	2.25	0.45
Specialization	2.27	0.65	2.08	0.76	1.73	0.30

^a^ : Productivity is noted in seconds and captures the handling time from start to finish and, when applicable, the time to repair errors.

^b^ : Production reliability is expressed in the proportion of products that were submitted without assembly flaws and took less than seven and a half minutes to produce (i.e., high production reliability).

^c^ : Motivational characteristic scores reflect a 5-point Likert scale. The higher the score, the more present the characteristic and the more likely that the operator can sustain the work.

*, 1 missing value; **, 4 missing values; ***, 3 missing values; ****, 2 missing values.

### 4.2 Human–cobot production unit 1 (limited job decision latitude)

In human–cobot production unit 1, operators could pause the cobot’s motion if they deemed it necessary. In total, 15 operators worked in this unit. Together, they produced 75 keyboards during both the manual and collaborative work sessions.

#### 4.2.1 Performance

**FIGURE 5:**
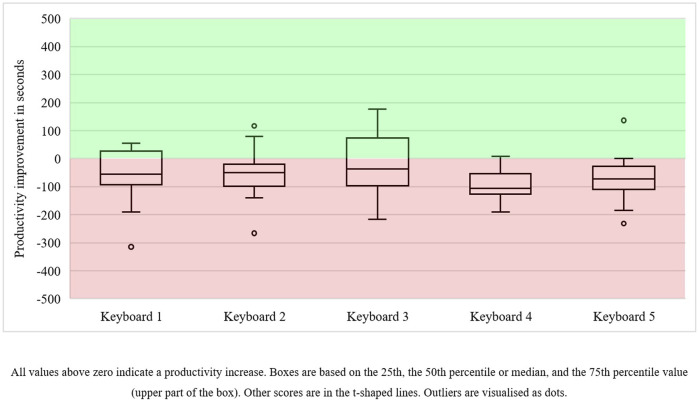
Productivity outcome improvements in operators in human–cobot production unit 1 (n = 15).

Production reliability improved marginally. We found that production reliability increased for 16 keyboards, while it deteriorated for 13 other keyboards—the latter were submitted on time during the manual work session but not during the collaborative session. Consequently, the number of keyboards with high production reliability increased from 46 in the manual work session to 49 keyboards in the collaborative work session (i.e., a six percent increase). Moreover, productivity only improved for 16 out of 75 keyboards (i.e., 21 percent). The minimal improvement in the operators’ productivity can be visualised using boxplots (Figure 5, presented after Table 2). They show that productivity decreased for most keyboards since all median scores, the black line going through the white boxes, are below 0—the zero line represents the manual productivity outcomes. Few improvements were found in keyboards 4 and 5, and less than a quarter of the operators achieved better productivity for keyboard 2. Most performance improvements can be found in keyboard 3, followed by keyboard 1.

Although the overall results in terms of both reliability and productivity were poor, we must emphasise that in this unit, the cobots’ tasks were fixed, as were its moderate speed and the programme it would run. This made the operator fully dependent on the cobot’s performance. Consequently, if the operator performed well manually, the cobot provided limited performance enhancement.

#### 4.2.2 Work perceptions

**FIGURE 6:**
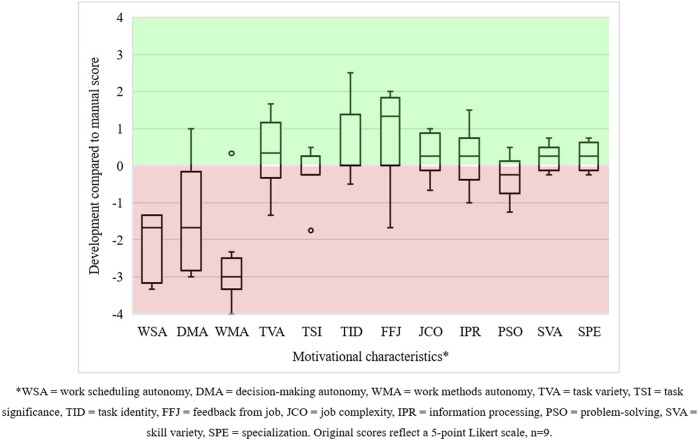
Development in the motivational characteristics of operators in human–cobot production unit 1.

Nine out of thirteen operators submitted valid work design questionnaires for both manual and collaborative work sessions, which allowed us to compare their scoring. The boxplots in Figure 6 show the difference between the motivational characteristics scores for both work sessions. Compared to manual production, all autonomy-related and problem-solving characteristics plummeted. We were not surprised by these results since operators had little job decision latitude and, therefore, limited opportunities to adapt the application of the cobot to their work preferences, losing their flexibility when working manually. One operator illustrated the rigidity of its human–cobot production unit as follows:

“*You are just working according to a certain protocol together with a certain [cobot] system. So yes, you would rather adapt to the system than vice versa, so to say*” (operator 13, unit 1).

The situation awareness assessments showed that nine operators passed all three error levels. All operators answered more than half of the error level 2 questions correctly, indicating that they understood the working situation they were in. Five out of fifteen operators failed error level 1, and four operators passed error level 3, while two operators failed both levels. These findings indicate that one-third of the operators had trouble sensing key elements in their working environment and predicting its near-future state. The fail rate for error level 1 surprised us since, with the obligatory use of the cobot and its moderate speed, the operators had ample opportunity to observe and predict the work situation. Failing these levels, however, showed that they did not. Six operators handled at least two out of three complacency checks correctly (i.e., not taking any faulty keys out of their sockets). The other nine operators failed most of their complacency tests. The high fail rate can be attributed to the cobot performing flawlessly and too slowly. Most operators tailed the cobot’s output and assumed that this output was correct, clearly focusing on other things. Consequently, they missed multiple times that the cobot presented incorrect keys.

After examining the operators’ performance and work perception outcomes in more detail, we found that only one operator in human–cobot production 1 realised higher performance and better work perception outcomes. This operator did not pause the cobot and made no requests for additional instrumental support. In contrast, three out of nine operators suffered lower performance and work perception outcomes. Most of these operators paused the cobot, requested additional instrumental assistance, failed error level 1, and handled most complacency checks incorrectly. We assume that the low job decision latitude provided to these operators largely contributed to these outcomes.

#### 4.2.3 Job decision latitudes, additional facilitating conditions, and human–cobot interdependencies

The job decision latitude in human–cobot production unit 1 was limited to pausing the cobot. This option was hardly ever used. Three operators paused the cobot once or twice to inspect their assembly work, verify the cobot’s output, or catch up with the cobot’s working pace. During the debriefing interviews, 10 operators mentioned that having only the option to pause the cobot was highly limited. These operators wanted to change the cobot’s speed and task allocation. As an example, operator 3 stated

“Every now and then, I thought to myself, that speed [of the cobot] could go up” (operator 3, unit 1).

Ten operators made twelve requests for additional instrumental assistance. Five requests were related to the cobot’s functioning. Operators checked with the workplace assistant to verify whether the cobot was functioning correctly or if they needed to use parts of cobot software, for which they had not received instructions. Seven requests were related to switching cobot modules (e.g., changing from barcode scanning to keyboard picking) or switching cobot programs (i.e., from keyboard type 1 to keyboard type 2). Three operators who experienced changeover issues made most of these requests and needed help activating the right cobot module or program. Apart from these incidents, all operators engaged in the predetermined human–cobot interdependence level (i.e., human–cobot interdependence level 4—the highest possible level) with limited additional instrumental assistance.

#### 4.2.4 Summary unit 1

In summary, operators in human–cobot production unit 1 barely used their available job decision latitude—it was not useful—and followed the cobot’s lead instead. Performance only improved for one-fifth of the keyboards. Although operators’ sense of autonomy plummeted, six other motivational characteristics received high scores from at least half of the operators. Signs of operator-out-of-the-loop behaviour became prevalent in the situation awareness assessments and complacency checks. Operators often paid insufficient attention to their current working environment and overestimated the environment’s future state. Moreover, many operators failed to respond accurately to repeated mistakes made by the cobot. The sustainability of this human–cobot production unit is questionable. In human–cobot production units where the human–cobot interdependence is predetermined and the cobot sets the working pace, operators get bored, focus their attention on other things, feel little autonomy, and perceive that the cobot largely dictates their performance. Initially, working with the new technology may be interesting, which is reflected in higher motivational characteristics scores than expected, but this novelty, we suspect, will soon wear off and leave the operator with a monotonous and rather dull machine assistant type of job.

### 4.3 Human–cobot production unit 2 (moderate job decision latitude)

In human–cobot production unit 2, operators could, next to pausing the cobot’s motion, also adjust the cobot’s speed and change its task allocation. The 13 operators in this unit produced 64 keyboards during the manual work sessions and 65 keyboards during the collaborative work sessions.

#### 4.3.1 Performance

**FIGURE 7:**
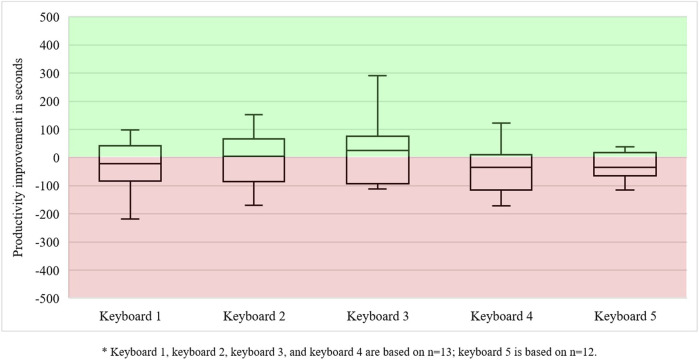
Productivity outcome improvements in operators in human–cobot production unit 2*.

Production reliability did not improve or deteriorate. Although eight out of 65 keyboards were produced with greater reliability during the collaborative work session, eight others were submitted too late. Productivity, however, improved for 28 out of 64 keyboards (i.e., 44 percent). However, we noticed that most performance improvements were concentrated amongst five operators with poor manual productivity. Overall, it was not a great result for performance in human–cobot production unit 2. However, learning how to use job decision latitudes in a new technological setup required time and clearly put production reliability and productivity under pressure. This can also be observed in Figure 7. Most operators could only increase their productivity for keyboards 2 and 3.

#### 4.3.2 Work perceptions

**FIGURE 8:**
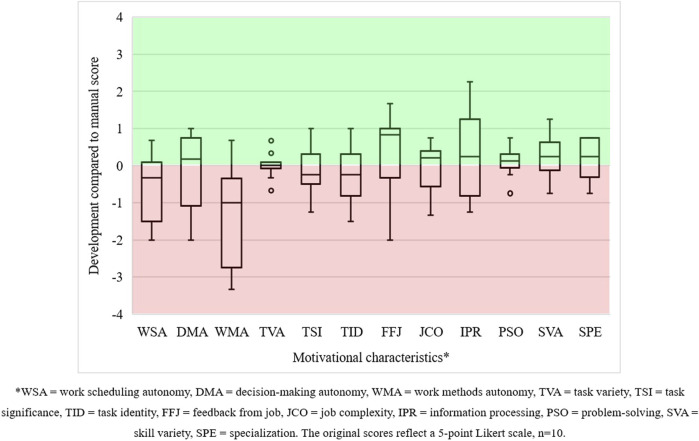
Development in the motivational characteristics of operators in human–cobot production unit 2.

We determined how the motivational characteristics developed for 10 of the 13 operators. Differences between their scores are visualised in Figure 8. For most of these operators, work scheduling autonomy, work method autonomy, task significance, and task identity decreased. In contrast, most scores for decision-making autonomy, feedback from the job, job complexity, information processing, problem solving, skill variety, and specialisation increased. Task variety scores remained unchanged.

The fact that work scheduling and work method autonomy decreased is less surprising than it seems. We attribute this to the cobot’s functioning and the organisation of the assembly work. The operator often had to wait for the cobot to finish. Meanwhile, there is very little else to be done. Nonetheless, the operator could change the production unit’s working pace and alter its task allocation. This likely sparked some sense of decision-making autonomy, more job complexity, and even some more information processing. All these indicators indeed increased, as did skill variety, as expected when having more job decision latitude in designing human–cobot task allocation. However, why task significance and task identity decreased is unclear. We must remember, however, to take the context into account as operator 7 confided

*“It is a very conveyor belt type of labour. I can imagine you can do this for two hours. But if you have to do this for an entire day, you would think ‘can I please get out of here?’”* (operator 7, unit 2).

The situation awareness assessments revealed that five operators passed all error levels, and all 13 passed error level 2. In contrast, five failed error level 1, and four failed error level 3, while one failed both levels. Moreover, we could assess how 11 operators handled the complacency checks. Only four of them handled most of the complacency checks correctly. The poor situation awareness and complacency results can be attributed to the cobot’s high operational speed, which the operators installed themselves. Consequently, operators had to play ‘catch-up with the cobot,’ paid little attention to what was (or could be) happening in their work environment, and failed to realise on time that some of the cobot’s output was incorrect.

Based on these outcomes, we found that one operator achieved better performance and work perception outcomes when working with the cobot. This operator performed poorly during their manual work session, increased the cobot’s operational speed for most keyboards, and did not request additional assistance. For two other operators, their performance and work perception deteriorated. These two operators also increased their cobot’s speed for most keyboards. However, they performed quite well during their manual work sessions and requested additional instrumental assistance. These operators are likely burdened by the cobot since they experienced difficulties operating it, hence the requested assistance, and seemed to have benefited only marginally from the cobot’s task performance due to their good manual performance. These insights stress that the operator’s manual outcomes and decision-making are important determinants of whether the human–cobot production unit can result in better outcomes. Both should be understood at an individual level.

#### 4.3.3 Job decision latitudes, additional facilitating conditions, and human–cobot interdependencies

We could observe that they used job decision latitudes for 61 keyboards. We found that all operators in this unit extensively utilised their job decision latitudes. Twelve out of 13 operators increased the cobot’s speed during 45 out of 61 keyboards. One operator, on the other hand, decreased the cobot’s speed while producing the first two keyboards. Moreover, 10 operators reallocated one or multiple tasks from the cobot to themselves. For 40 out of 61 keyboards, the cobot was used for fewer tasks. Operators considered themselves faster at ‘pick and verify’ tasks and used the cobot for the more cognitive tasks (i.e., searching and inspecting). In exceptional cases, the operator collected the keys or performed final inspections. For 30 keyboards, the “task allocation” and “speed adjustment” job decision latitudes were used simultaneously, impacting both the human–cobot interdependence’s level and dynamics.

Six operators made eight requests for additional instrumental assistance. Except for one question about task allocation requirements, they pertained mainly to switching cobot modules and programs. Operators asked these questions to reassure themselves and prevent themselves from making mistakes when switching between modules or programs. No changeover errors by operators occurred in production unit 2. It remained unclear whether more operators wanted additional instrumental assistance but decided not to ask questions to save time and thus achieve better performance.

The extensive use of job decision latitudes drastically affected the human–cobot interdependence level. We were able to determine these levels for 56 keyboards. The operator and cobot engaged in interdependence level 2 to produce 24 keyboards. Nine were produced with human–cobot interdependence level 3. For another 18 keyboards, 7 operators decided to keep their human–cobot interdependence at level 4 (maximum). Finally, by only using the cobot to collect the keys, one operator resorted to human–cobot interdependence level 1 for producing all five keyboards. Due to the speed adjustments, the dynamics of 45 human–cobot interdependencies increased. For two others, it decreased.

#### 4.3.4 Summary unit 2

In summary, operators in this unit used the decision options available to them and started to redesign their human–cobot interdependence. They were willing and able to adjust their interdependencies without much additional assistance. However, using these job decision latitudes frequently led to high work pressure. Some operators decided to lower their human–cobot interdependence level to 2, whereas others kept it at level 4 but increased their cobot’s speed considerably. This stresses the variation in design opportunities that emerge when there are more job decision latitudes to use. It is also apparent that situational awareness and complacency are under pressure when human–cobot interdependence becomes more dynamic. Operators were unaware of their work environment and handled cobot-related flaws poorly. Remarkably, operators seemed mainly focused on reducing time but seemed unaware of the high-strain working conditions that they, in the meantime, created for themselves.

### 4.4 Human–cobot production unit 3 (high job decision latitude)

In human–cobot production unit 3, 12 operators could, next to pausing the cobot, adjusting the cobot’s speed, and allocating tasks, also change the buildup of the cobot’s program by adding, removing, or changing commands.

#### 4.4.1 Performance

**FIGURE 9:**
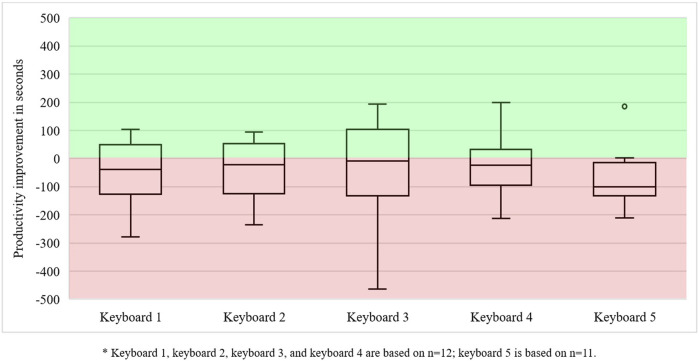
Productivity outcome improvements in operators in human–cobot production unit 3*.

Performance data for 59 out of 60 keyboards were obtained. The data indicate that production reliability decreased. Only 7 out of 59 keyboards were produced with higher production reliability. However, the production reliability decreased for 11 keyboards since they were no longer submitted on time. Consequently, the number of keyboards with high production reliability decreased by nine percent. Productivity improved for 21 keyboards (i.e., a 36 percent increase). For the other 38 keyboards, productivity considerably decreased. As observed in Figure 9 (next page), we encountered large differences in productivity development.

Figure 9 shows that most keyboards were produced slower. Although almost all operators failed to improve their productivity for keyboard 5, considerably more operators were able to substantially improve their productivity outcomes for the other keyboards. The length of the boxplots (i.e., the difference between the highest and lowest values) shows that some operators achieved considerable productivity gains, while others suffered great productivity losses. Trying to change the buildup of their cobot programs and using moderately complex job decision latitudes, very fast or slow manual performance, making mistakes, and requesting assistance have all contributed to these big differences. This, again, stresses the importance of carefully looking into the individual differences between operators.

#### 4.4.2 Work perceptions

**FIGURE 10:**
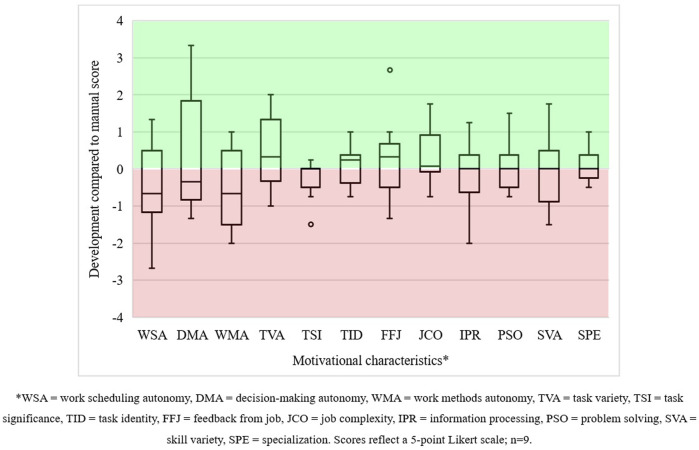
Development in the motivational characteristics of operators in human–cobot production unit 3.

Nine out of 12 operators submitted valid work design questionnaires. Differences between their scores are visualised in Figure 10. Autonomy-related characteristics decreased for many operators. Moreover, the stretched boxplots of these characteristics illustrate that some operators perceived considerably more autonomy than others. Task variety, task identity, feedback from the job, and job complexity improved for most operators. A clear differentiation between the operators’ scoring is visible for the other characteristics.

From these results, we learn that high job decision latitude can help operators preserve their motivational characteristics and, for some, even improve them strongly. However, for some, this level of decision latitude can be too much of a good thing and lead to a loss of perceived autonomy and a degradation of other work characteristics.

The situation awareness assessments showed that 8 out of 12 operators passed all error levels. Four operators failed error level 1, and one failed error level 3. Moreover, we found that 8 out of 11 operators handled 2 complacency checks correctly. We attribute these outcomes to the high amount of available job decision latitudes. On one hand, by being given the ability and encouragement to dive deeper into the cobot’s programs, operators may have, consequently, paid enough mental attention to the cobot and the work environment to pass the situation awareness assessments and complacency checks. On the other hand, operators used their job decision latitude to remove the faulty key collection from their cobot program. Therefore, the cobot handled most complacency checks successfully.

Based on the outcome data, we found that two operators achieved better performance and work perception outcomes. These operators decreased their human–cobot interdependence level, increased their cobot’s operational speed, primarily achieved productivity improvements, and passed all situation awareness assessments—one of them changed the cobot’s programs. In contrast, three other operators hardly achieved any performance improvements and no work perception improvement. These operators used their job decision latitudes quite differently. One decreased the cobot’s speed and made an assisted attempt at changing the cobot’s programs, while another increased the cobot’s speed and left the cobot programs intact. We thus see highly individual use of decision latitudes with very different results in this production unit.

#### 4.4.3 Job decision latitudes, additional facilitating conditions, and human–cobot interdependencies

We could determine which job decision latitudes were used to produce 54 keyboards. The cobot’s speed and task allocation were changed most frequently. A total of 10 operators adjusted the cobot’s speed during the production of 47 keyboards. Nine operators increased the cobot’s speed for four or five keyboards, and one operator decreased the cobot’s speed for all five. In total, the task allocation for 38 keyboards was adjusted. For 36 of these keyboards, the operator checked the barcode. Moreover, in 21 instances, the operator picked the keyboard. Finally, nine keyboards were inspected by the operator. The cobot always collected the keys. The cobot’s motion was paused once.

In addition to changing these job decision latitudes, half of the operators changed the buildup of at least one program. These changes comprised the following: a reduction in halts between the cobot modules, the removal of faulty key picking (i.e., the complacency check), the removal or suppression of the program structure, and the addition of extra cobot movements (i.e., waypoints). The adjustments directly impacted the reliability and duration of the cobot programs. Moreover, three operators suggested relevant program changes that were, unfortunately, technically unfeasible during the work session. Six operators stated that they deliberately did not change their cobots’ program due to a sense of time pressure caused by the demand to submit an error-free keyboard every seven and a half minutes. Operator 10 illustrated this as follows:

*“At a sudden moment, I thought to myself, ‘oh, I want to briefly check how everything [the cobot program] works.’ But then I thought, ‘oh, I have to finish that thing [the keyboard] in seven-and-a-half minutes’. … Then I thought, ‘I will continue [producing the keyboard]”* (operator 10, unit 3).

Job decision latitudes were combined frequently. Thirty keyboards came with adjusted task allocations and speeds. For 11 keyboards, the tasks, cobot speed, and cobot programs were changed, resulting in different human–cobot interdependence levels and unique dynamics.

Nine operators made 22 requests for additional instrumental support. Ten of these requests were made by six operators and were related to changing the buildup of one or more cobot programs. The operators either verified the feasibility of their programming ideas with the workplace assistant or sought assistance to change the cobot’s program buildup. The importance of available workplace assistance became clear when two operators changed the buildup of their cobot program on their own. Their programming efforts failed and resulted in faulty and less predictable cobot applications (i.e., missing keys or the cobot halting at unforeseen places). The relevance of having additional instrumental assistance to fall back on was stressed by operator 5 as follows:

*“I knew what I was doing, and if things went wrong, I asked you or someone else what to do. The assistance was good”* (operator 5, unit 3).

We found that 17 out of 54 keyboards were produced at interdependence level 4. Another 18 keyboards were produced at level 2, primarily using the cobot to collect the keys and conduct final inspections. Interdependence level 3 was used to produce 13 keyboards. The operator performed the barcode scanning task in all but one case. Finally, six keyboards were produced with human–cobot interdependence level 1. The cobot was only used to collect the keys, while the operator performed all other tasks. The dynamics of 41 human–cobot interdependencies changed considerably.

#### 4.4.4 Summary unit 3

In summary, we are impressed with the effort the operators invested in changing the buildup of their cobot programs. It showed that they can do so if good assistance is available. However, the conflict between achieving superior performance and investing time in exploring and optimising cobot programs also became apparent. Some operators stuck to their primary goal of achieving better performance and ignored their opportunity to change the cobot’s programs. Our strict performance targets may have triggered this decision. This underlines the importance of contextualising opportunities to cope with time pressure (e.g., some unaccounted time to change the cobot’s programs). Furthermore, it seemed that human–cobot production unit 3 triggered a winner-takes-all effect. Although some operators achieved better performance and work perception outcomes, both decreased strongly for others. Their alertness was remarkable and outstanding, suggesting that more complex job decision latitudes keep operators alert and engaged. Despite the data being inconclusive about whether changing the cobot’s program holds the key to superior performance and work perceptions, we gained a valuable insight into how inexperienced cobot operators used such an advanced job decision latitude, the risks it entails, and what they need.

### 4.5 Overview

Table 3 (next page) summarises the key insights of this results section. This overview shows that each type of human–cobot production unit captures the requirements, benefits, and consequences of different job decision latitudes. We will use these insights in the next section to finalise our research.

**TABLE 3:** Overview of key insights per human–cobot production unit.

Core theme	Human–cobot production unit		
	Unit 1 (n = 15)	Unit 2 (n = 13)	Unit 3 (n = 12)
Job decision latitude			
Job decision latitudes leveraged by at least half of the operators	None	Speed adjustment Task reallocation	Speed adjustment Task reallocation Program alteration
Additional instrumental assistance			
Requests for additional instrumental assistance	12 requests by 6 operators	8 requests by 6 operators	22 requests by 9 operators
Human–cobot interdependence			
Dominant human–cobot interdependence level	4: cobot performs all tasks	2: cobot collects and inspects	2: cobot collects and inspects
Performance outcomes			
Change in the portion of keyboards with high production reliability	+7% Missing: 0	0% Missing: 0	−9% Missing: 1
Keyboards with a productivity improvement	16/75 (21%) Missing: 0	28/64 (44%) Missing: 1	21/59 (36%) Missing: 1
Work perception outcomes			
Motivational characteristics that received a higher score by at least half of the operators	6/12 (50%) Missing: 6	7/12 (58%) Missing: 3	4/12 (33%) Missing: 3
Operators that passed all situation awareness error levels	9/15 (60%) Missing: 0	5/13 (38%) Missing: 0	8/12 (67%) Missing: 0
Operators that handled most complacency checks successfully	6/15 (40%) Missing: 0	4/11 (36%) Missing: 2	8/11 (72%) Missing: 1

## 5 Discussion

In this last section, we draw our conclusions and discuss this study’s implications, limitations, and opportunities for future research.

### 5.1 Conclusion

This research aimed to richly describe the use of job decision latitude by individual operators, and its implications for the design of human-cobot interdependencies and the outcomes of human-cobot production units. To achieve this goal, the following two research questions were formulated: 1. to what extent can and will individual operators autonomously use their available job decision latitudes to design human–cobot interdependencies? 2. a. How does using job decision latitudes change the design of human–cobot interdependencies? and b. do they achieve better performance and work perception outcomes than a manual work system? After creating a rigorous research setting; running 40 simulations with student participants; and conducting observations, assessments, surveys, and debriefing interviews, we obtained an in-depth understanding of the use and implications of job decision latitude for human–cobot production units in an artificial high-mix low-volume production context. We can now answer our questions at a general level.

1. Operators in this research, mainly technical bachelor’s students, were largely capable of autonomously using the different job decision latitudes we provided them with. Changing commands in cobot programs, however, required considerably more additional instrumental assistance. The latter indicates that we exceeded the limit of what we could ask from our inexperienced and crash-course-trained operators. Only excessive workplace assistance could compensate for their lack of capacity. Regarding the operators’ willingness to use their available job decision latitudes, we learnt that operators deliberately used or ignored them. Job decision latitudes were mainly used if they would contribute to faster production (i.e., time reduction) rather than making work more sustainable.
2a. Using job decision latitudes changed the human–cobot interdependence levels. These were mostly lower than those of predetermined human–cobot production units. Moreover, in most cases, the cobot’s speed was increased, which intensified the dynamics of the human–cobot interdependencies. Some operators frequently used the possibility of program change, which resulted sometimes, but definitely not always, in better functioning human–cobot interdependence. In exceptional cases, the human–cobot interdependence became unstable.
2b. Concerning outcomes, we found that productivity strongly improved in human–cobot production units with more job decision latitudes. This is likely due to the increased cobot speed and the reallocation of tasks that the operator could execute faster. Production reliability, on the other hand, did not improve in the units because of the time the operators needed to use their job decision latitudes. This was no issue for operators in the predetermined human–cobot production units since they achieved more production reliability. Although higher job decision latitude does not automatically lead to more extensive improvements in motivational characteristics, it simulates the operator’s sense of autonomy, which, given the additional decision-making opportunities, makes sense. Moreover, operator-out-of-the-loop behaviour is the least likely when job decision latitude is high. Operators who are challenged to familiarise themselves with the cobot’s functioning and actively try to make cobot programs more robust showed the best situational awareness and the least automation-induced complacency. Finally, since they primarily used their job decision latitudes to achieve better performance, operators burdened themselves with more work demands and made their work less sustainable.

These conclusions illustrate the complexity of providing operators with job decision latitudes to design their own human–cobot interdependencies and create human–cobot production units that improve performance and work perceptions. Based on our results, we conclude that a ‘one-size-fits-all’ approach does not work. While some operators performed poorly during the manual work sessions and benefitted from almost any form of human–cobot interdependence, others needed minimal assistance and were mostly hindered by the cobot when they lacked the job decision latitudes to redesign their unit. Furthermore, operators appreciated the availability of job decision latitudes differently. While some experienced too few job decision latitudes, which are known to facilitate poor working conditions (Karasek, 1979), others experienced ‘too much of a good thing’ (Langfred, 2004; Zhou, 2020) and deliberately decided to ignore some job decision latitudes despite their potential. These insights stress the importance of a tailored approach and the need to take the context into account.

Regarding the context, when the amount and complexity of job decision latitudes increase, the contextualisation of workplace resources becomes more important for the operator to dominate the cobot’s application. These resources are important not only to respond to the operator’s calls for assistance and motivate the operator to use their job decision latitudes but also—and more importantly—to prevent the operator from making dangerous and unsustainable design decisions. Parker et al. (2019) referred to this phenomenon as “poor work design begets poor design” (p. 907). This requires a solid alignment between the tasks at hand, the robustness of the cobot technology, the available job decision latitudes, and the contextualised workplace resources. Such alignment allows for more dynamic designs and favours integrating the human–robot interaction field with that of MSTS.

### 5.2 Theoretical implications

In this work, we raised awareness for a deep-rooted engineering bias in human–robot interaction research that systematically strives toward the functional but highly rigid design of human–cobot production units that the operator cannot change (El Zaatari et al., 2019; Hentout et al., 2019). Such designs fail to adapt to changing work perceptions and work demands and foster unsustainable work and limited performance. To make the design of human–cobot production units more dynamic, we proposed to provide operators with job decision latitudes to design their human–cobot interdependencies and were the first to study how operators navigated these opportunities and what this meant for design and outcomes. Creating a cross-over between human–robot interaction literature and MSTS—more recently also referred to as workplace innovation (Oeij et al., 2023)—results in theoretical implications for both streams.

Our work provides human–robot interaction engineers with empirical evidence that operators can and want to play a more prominent role in designing their human–cobot interdependence and found that their efforts bear the potential to make human–cobot product units more useful and sustainable; some operators, however, could benefit from more support that translates the impact of certain design-related decisions (Pammer et al., 2017; Parker et al., 2019). Therefore, adding the MSTS concept of job decision latitude to human–robot design methodology, such as Johnson’s (2014) coactive design method, offers a way to establish more sophisticated designs that take not only the technical but also individual human preferences and changing work demands into account (Reiman et al., 2023). Our work facilitates this incorporation in two main ways.

On one hand, we offered insight into the technical and organisational resources that an individual operator needs to design and redesign their human–cobot interdependencies safely. More job decision latitudes require increasingly robust cobot technology and expert workplace assistance. Moreover, pausing the cobot’s motion is not enough. The operator must directly influence the level and dynamics of their human–cobot interdependence through task, speed, and command adjustments. In this respect, our work is exemplary since the cobot studies we know about only use pre-programmed cobots or provide a very limited number of configurations to choose from (Bauer et al., 2016).

On the other hand, we provided a more comprehensive approach to assess the outcomes of human–cobot production units. We were able to combine traditional performance measures with measures from applied psychology. We deliberately used the traditional motivational characteristics (Morgeson and Humphrey, 2006; Humphrey et al., 2007) and operator-out-of-the-loop measures (Endsley, 1988; Parasuraman and Manzey, 2010) to assess the sustainability of human–cobot production units, which, to the best of our knowledge, has never been done before. This combination could prevent design methods from striving toward designs with a highly economic rationale but unsustainable work. Incorporating applied psychology measures in human–robot interaction methodology is, therefore, of essence.

Our work also contributes to the MSTS literature. First, we were able to successfully apply a core, if not the most prominent, MSTS design principle of sufficient job decision latitude at the operational level in a new context (i.e., the development of human–cobot production units). This uncovered a promising avenue for future MSTS research. Second, our findings contribute to democratising work design (Krogstie et al., 2013; Guest et al., 2022). Not only do we gain an initial understanding of when operators experience either insufficient job decision latitudes or an excess of them, but it also becomes clear how providing operators with more job decision latitudes impacts the design and the outcomes of production units. These insights empirically support the democratisation of work design but stress the need for a tailored approach toward operator involvement (Panagou et al., 2024).

### 5.3 Practical implications

This research captures a manufacturing technology that is gaining popularity (International Federation of Robotics, 2023) and provides three contributions to manufacturing practice.

First is the business case behind the democratisation of work design. The three human–cobot production units under study provide different perspectives and reveal the dynamics between flexibility and affordability. Unit 1 is the least flexible but most affordable one. The operator works according to a fixed procedure, and minimal investments are required to operate the cobot and achieve performance increases. However, due to the operators’ low sense of autonomy and their tendency to show operator-out-of-the-loop behaviour, it is unlikely that these operators can sustain their work or respond to changes in the work environment. Unit 3 is on the other end of the spectrum (i.e., highly flexible but the least affordable). In this situation, the operator fulfils the role of cobot operator and partially the role of cobot engineer. High investments are required to prepare the operator for this role, which, so far, has not always resulted in better performance and work perceptions. Unit 2 balances somewhere in the middle. With its modular cobot programs and moderate investment in instructions and support, such a unit is quite flexible and affordable. However, it is important to check whether the operators have burdened themselves with too many tasks or an excessively rapid working pace since both are likely to suppress the unit’s sustainability.

Second is the expanding role of the engineer. We argue that the engineering role should be more concerned with establishing sustainable work and working conditions. The engineer should converse with the operator about their working preferences and desired robotic support and convert these inputs into modular cobot applications. Moreover, in collaboration with production management, the engineer must ensure that the operator makes constructive decisions regarding the work design (i.e., decisions that not only enhance performance but also ensure sustainable work). Consequently, the engineer must not only train the operator to safely work with the cobot but also help the operator understand the consequences of their design decisions and direct the operator toward alternative design options if necessary. Having a clear understanding of the instrumental assistance required by the operator is key. Moreover, workplace resources, such as a helpdesk or chatbot, should be organised to support the operator’s need for additional instrumental assistance. Engineers who want to become more familiar with sustainable work should contact their HR department since these professionals will likely be most knowledgeable about this topic.

Third is the complexity involved in developing workstations that allow operators to design their human–cobot interdependencies. It took us 6 months and a tremendous amount of time to sketch, build, test, and solidify the workstations used for this study. Nevertheless, their robustness was far from ideal. Our key insight in this respect is that the more complex job decision latitudes are embedded in the workstation, the more degrees of freedom an operator has to design their human–cobot interdependence, and the more robust the workstation must be to safely and technically process these decisions. Therefore, we advise engineers to first design workstations with modular cobot applications and gradually proceed toward workstations that also allow the operator to add and remove cobot-related commands. This way, it might take less time to build functional human–cobot production units.

### 5.4 Limitations and future research

In this subsection, we highlight four major limitations and equip these with suggestions for future research.

First, considering the complexity of the theory, the sample of n = 40 was still relatively small. Although we would maintain that the differences between the operators are large enough for our descriptive study to show the differences in the use of job decision latitudes and some possible effects, a follow-up study where the differences can be tested further on significance is certainly necessary. This would directly adhere to the guidelines described by Parker and Grote (2022) and Weiss et al. (2021), who urge for a better understanding of how individuals make work design choices and how satisfying working conditions for humans and productivity-enhancing cobots can be created through so-called “co-shaping.”

Second, in our work, we did not account for the operators’ individual characteristics (e.g., level of education, affiliation with technology, personality, and working experience). It seems relevant to research which operators’ characteristics could predict optimal decision latitude levels as we observed large individual differences within units. This could, again, be achieved in a laboratory setting or a highly structured yet flexible production system in a real industrial environment—the latter would also directly feed into the call for more MSTS research in contemporary manufacturing settings (Govers and van Ameldvoort, 2023). Researchers should include operators with distinct characteristics to best study between-subject differences, particularly individuals representing the target audience (i.e., employed operators).

Third, we noticed that time pressure influenced the operators’ use of job decision latitudes. Removing the timer and prioritising work perceptions over performance outcomes could reduce this sensation and potentially result in different outcomes. Nonetheless, future research endeavours are recommended to control for time pressure using the time pressure component, as suggested by Shukla and Srivastava (2016).

Fourth, we applied our own concept of human–cobot interdependence levels to characterise the interaction between the operator and the cobot. We acknowledge that this typology lacks depth and provides limited insight into whether the operator is helped or hindered by the cobot at a task level. The coactive design method by Johnson (2014) offers a more extensive typology, but this proved unsuitable for this research because it was unclear how to relate the typology to the operator and the cobot. Therefore, we stress the need for an assessment technique that could indicate the extent to which the cobot’s potential has been embedded in the design of human–cobot interdependence. We will work on a proposed solution and revisit this in our future work.

## Data Availability

The datasets presented in this article are not readily available because respondents were assured their data would remain confidential and would not be shared. Requests to access the datasets should be directed to m.r.wolffgramm@saxion.nl.

## References

[B1] Abbad M. M. M. (2021). Using the UTAUT model to understand students’ usage of e-learning systems in developing countries. Educ. Inf. Technol. 26 (6), 7205–7224. 10.1007/s10639-021-10573-5 PMC8122219 34025204

[B2] Ali R. Islam T. Prato B. R. Chowdhury S. Al Rakib A. (2023). Human-Centered design in human-robot interaction evaluating user experience and usability. Bull. Bus. Econ. (BBE) 12 (4), 454–459. 10.61506/01.00148

[B3] Baltrusch S. J. Krause F. de Vries A. W. van Dijk W. de Looze M. P. (2022). What about the human in human robot collaboration? Ergonomics 65 (5), 719–740. 10.1080/00140139.2021.1984585 34546152

[B4] Bauer W. Bender M. Braun M. Rally P. Scholtz O. (2016). “Lightweight robots in manual assembly - best to start simply,” in Examining companies' initial experiences with lightweight robots. Stuttgart: Fraunhofer IAO, Stuttgart.

[B5] Benders J. Hoeken P. Batenburg R. Schouteten R. (2006). First organise, then automate: a modern socio-technical view on ERP systems and teamworking. New Technol. work, Employ. 21 (3), 242–251. 10.1111/j.1468-005x.2006.00178.x

[B6] Berkers H. A. Rispens S. Le Blanc P. M. (2023). The role of robotization in work design: a comparative case study among logistic warehouses. Int. J. Hum. Resour. Manag. 34 (9), 1852–1875. 10.1080/09585192.2022.2043925

[B7] Bringes C. Lin Y. Sun Y. Alqasemi R. (2013). Determining the benefit of human input in human-in-the-loop robotic systems. IEEE, 210–215. 10.1109/roman.2013.6628447

[B8] Calitz A. P. Poisat P. Cullen M. (2017). The future African workplace: the use of collaborative robots in manufacturing. SA J. Hum. Resour. Manag. 15 (1), 1–11. 10.4102/sajhrm.v15i0.901

[B10] Çençen A. (2019). Adaptable framework methodology for designing human-robot coproduction. Delft: TU Delft.

[B11] Chou R. J. Robert S. A. (2008). Workplace support, role overload, and job satisfaction of direct care workers in assisted living. J. health Soc. Behav. 49 (2), 208–222. 10.1177/002214650804900207 18649503

[B12] Clark H. H. (1996). Using language. 1. publ. edn. Cambridge: Cambridge Univ. Press.

[B13] Coronado E. Kiyokawa T. Ricardez G. A. G. Ramirez-Alpizar I. G. Venture G. Yamanobe N. (2022). Evaluating quality in human-robot interaction: a systematic search and classification of performance and human-centered factors, measures and metrics towards an industry 5.0. J. Manuf. Syst. 63, 392–410. 10.1016/j.jmsy.2022.04.007

[B14] de Sitter L. U. den Hertog J. F. Dankbaarl B. (1997). From complex organizations with simple jobs to simple organizations with complex jobs. Hum. Relat. (New York) 50 (5), 497–534. 10.1177/001872679705000503

[B15] Dhondt S. Delano Pot F. O. Kraan K. (2014). The importance of organizational level decision latitude for well-being and organizational commitment. Team Perform. Manag. 20 (7/8), 307–327. 10.1108/tpm-03-2014-0025

[B16] Draghi M. (2024). The future of European competitiveness Part A: a competitiveness strategy for europe. Italy: European Commission.

[B17] Eaton D. R. (2004). *Improving the management of reliability*, acquisition research program. Monterey: Navel Postgraduate School.

[B18] El Makrini I. Elprama S. A. Van den Bergh J. Vanderborght B. Knevels A. Jewell C. I. C. (2018). Working with walt: how a cobot was developed and inserted on an auto assembly line. IEEE robotics and automation Mag. 25 (2), 51–58. 10.1109/mra.2018.2815947

[B19] El Zaatari S. Marei M. Li W. Usman Z. (2019). Cobot programming for collaborative industrial tasks: an overview. Robotics Aut. Syst. 116, 162–180. 10.1016/j.robot.2019.03.003

[B20] Endsley M. R. (1988). “Situation awareness global assessment technique (SAGAT),” in *Proceedings of the IEEE 1988 national aerospace and electronics conference*IEEE, 789.

[B21] Endsley M. R. Garland D. J. (2000). Theoretical underpinnings of situation awareness: a critical review. Situat. Aware. analysis Meas. 1 (1), 3–21.

[B22] Endsley M. R. Kiris E. O. (1995). The out-of-the-loop performance problem and level of control in automation. Hum. factors 37 (2), 381–394. 10.1518/001872095779064555

[B23] European Commission (2024). ERA industrial technologies roadmap on human-centric research and innovation for the manufacturing sector. Luxembourg: Publications Office of the European Union.

[B24] Falk A. Heckman J. J. (2009). Lab experiments are a major source of knowledge in the social sciences. Science 326 (5952), 535–538. 10.1126/science.1168244 19900889

[B25] Fenlason K. J. Beehr T. A. (1994). Social support and occupational stress: effects of talking to others. J. Organ. Behav. 15 (2), 157–175. 10.1002/job.4030150205

[B26] Gasteiger N. Ahn H. S. Lee C. Lim J. MacDonald B. A. Kim G. H. (2022). Participatory design, development, and testing of assistive health robots with older adults: an international four-year project. ACM Trans. human-robotic Interact. 11 (4), 1–19. 10.1145/3533726

[B27] Goujon A. Rosin F. Magnani F. Lamouri S. Pellerin R. Joblot L. (2024). Industry 5.0 use cases development framework. Int. J. Prod. Res. 62, 6064–6089. 10.1080/00207543.2024.2307505

[B28] Govers M. Amelsvoort P. V. (2019). A socio-technical perspective on the digital era: the lowlands view. Eur. J. Workplace Innovation 4 (2). 10.46364/ejwi.v4i2.589

[B29] Govers M. van Amelsvoort P. (2023). A theoretical essay on socio-technical systems design thinking in the era of digital transformation. Gruppe. Interakt. Organ. Z. für angew. Organ. 54 (1), 27–40. 10.1007/s11612-023-00675-8

[B30] Guest D. Knox A. Warhurst C. (2022). Humanizing work in the digital age: lessons from socio-technical systems and quality of working life initiatives. Hum. Relat. (New York) 75 (8), 1461–1482. 10.1177/00187267221092674

[B34] Hentout A. Aouache M. Maoudj A. Akli I. (2019). Human-robot interaction in industrial collaborative robotics: a literature review of the decade 2008-2017. Adv. Robot. 33 (15-16), 764–799. 10.1080/01691864.2019.1636714

[B35] Huang H. He Y. Li D. (2018). Coordination of pricing, inventory, and production reliability decisions in deteriorating product supply chains. Int. J. Prod. Res. 56 (18), 6201–6224. 10.1080/00207543.2018.1480070

[B36] Humphrey S. E. Nahrgang J. D. Morgeson F. P. (2007). Integrating motivational, social, and contextual work design features: a meta-analytic summary and theoretical extension of the work design literature. J. Appl. Psychol. 92 (5), 1332–1356. 10.1037/0021-9010.92.5.1332 17845089

[B37] International Federation of Robotics (2023). World robotics 2023 report Asia ahead of Europe and the Americas. Frankfurt: Normans Media Ltd.

[B39] Johansen K. Rao S. Ashourpour M. (2021). The role of automation in complexities of high-mix in low-volume production – a literature review. Procedia CIRP 104, 1452–1457. 10.1016/j.procir.2021.11.245

[B40] Johnson M. (2014). Coactive design: designing support for interdependence in human-robot teamwork. Delft: TU Delft.

[B41] Jones D. G. Endsley M. R. (1996). Sources of situation awareness errors in aviation. Aviat. space, Environ. Med. 67 (6), 507–512. 8827130

[B42] Karasek R. A. (1979). Job demands, job decision latitude, and mental strain: implications for job redesign. Adm. Sci. Q. 24 (2), 285–308. 10.2307/2392498

[B43] Krogstie B. Pammer V. Prilla M. (2013). Fostering collaborative redesign of work practice: challenges for tools supporting reflection at work. Limited, United Kingdom: Springer London.

[B44] Kuipers H. Van Amelsvoort P. Kramer E. (2020). New ways of organizing: alternatives to bureaucracy. 1st edn. Leuven: Acco Uitgeverij.

[B45] Laine E. Malm T. Latokartano J. (2007). Human and industrial robot cooperation – technologies and practical examples, 397. *SLAS 2007* .

[B46] Langfred C. W. (2004). Too much of a good thing? Negative effects of high trust and individual autonomy in self-managing teams. Acad. Manag. J. 47 (3), 385–399. 10.2307/20159588

[B47] Merat N. Seppelt B. Louw T. Engström J. Lee J. D. Johansson E. (2019). The “Out-of-the-Loop” concept in automated driving: proposed definition, measures and implications. Cognition, Technol. and work 21 (1), 87–98. 10.1007/s10111-018-0525-8

[B48] Merritt S. M. Ako-Brew A. Bryant W. J. Staley A. McKenna M. Leone A. (2019). Automation-induced complacency potential: development and validation of a new scale. Front. Psychol. 10, 225. 10.3389/fpsyg.2019.00225 30837913 PMC6389673

[B49] Morgeson F. P. Humphrey S. E. (2006). The Work Design Questionnaire (WDQ): developing and validating a comprehensive measure for assessing job design and the nature of work. J. Appl. Psychol. 91 (6), 1321–1339. 10.1037/0021-9010.91.6.1321 17100487

[B50] Muschalla B. Henning A. Haake T. W. Cornetz K. Olbrich D. (2020). Mental health problem or workplace problem or something else: what contributes to work perception? Disabil. rehabilitation 42 (4), 502–509. 10.1080/09638288.2018.1501099 30451011

[B51] Oeij P. R. A. Dhondt S. (2024). Reviewing workplace innovation as a plea for a practical approach. Sociol. compass 18 (4). 10.1111/soc4.13203

[B52] Oeij P. R. A. Dhondt S. McMurray A. J. (2023). “Workplace innovation: a converging or diverging research field?,” in A research agenda for workplace innovation (Cheltenham, UK: Edward Elgar Publishing), 201–252.

[B53] Ore F. Hansson L. Wiktorsson M. (2017). Method for design of human-industrial robot collaboration workstations. Procedia Manuf. 11, 4–12. 10.1016/j.promfg.2017.07.112

[B54] Pammer V. Krogstie B. Prilla M. (2017). Let's talk about reflection at work. Int. J. Technol. Enhanc. Learn. 9 (2-3), 151–168. 10.1504/ijtel.2017.084493

[B55] Panagou S. Neumann W. P. Fruggiero F. (2024). A scoping review of human robot interaction research towards Industry 5.0 human-centric workplaces. Int. J. Prod. Res. 62 (3), 974–990. 10.1080/00207543.2023.2172473

[B56] Parasuraman R. Manzey D. H. (2010). Complacency and bias in human use of automation: an attentional integration. Hum. factors 52 (3), 381–410. 10.1177/0018720810376055 21077562

[B57] Parasuraman R. Molloy R. Singh I. L. (1993). Performance consequences of automation-induced'complacency. Int. J. Aviat. Psychol. 3 (1), 1–23. 10.1207/s15327108ijap0301_1

[B58] Parasuraman R. Sheridan T. B. Wickens C. D. (2000). A model for types and levels of human interaction with automation. IEEE Trans. Syst. man, cybernetics-Part A Syst. Humans 30 (3), 286–297. 10.1109/3468.844354 11760769

[B59] Parker S. K. Andrei D. M. Van den Broeck A. (2019). Poor work design begets poor work design: capacity and willingness antecedents of individual work design behavior. J. Appl. Psychol. 104 (7), 907–928. 10.1037/apl0000383 30640488

[B60] Parker S. K. Boeing A. A. (2023). “Workplace innovation in the digital era: a role for SMART work design,” in A research agenda for workplace innovation (Cheltenham, UK: Edward Elgar Publishing), 91–112.

[B61] Parker S. K. Grote G. (2022). Automation, algorithms, and beyond: why work design matters more than ever in a digital world. Appl. Psychol. 71 (4), 1171–1204. 10.1111/apps.12241

[B62] Pilat D. Schreyer P. (2002). Measuring productivity. OECD Econ. Stud. 33 (2), 127–170. 10.1787/eco_studies-v2001-art13-en

[B63] Prati E. Peruzzini M. Pellicciari M. Raffaeli R. (2021). How to include user eXperience in the design of human-robot interaction. Robotics computer-integrated Manuf. 68, 102072. 10.1016/j.rcim.2020.102072

[B64] Reiman A. Kaivo-oja J. Parviainen E. Takala E. Lauraeus T. (2023). Human work in the shift to Industry 4.0: a road map to the management of technological changes in manufacturing. Int. J. Prod. Res. 62, 5613–5630. 10.1080/00207543.2023.2291814

[B65] Renda A. Schwaag Serger S. Tataj D. Morlet A. Isaksson D. Martins F. (2021). Industry 5.0, a transformative vision for Europe. Luxembourg: Publications Office.

[B66] Rogers M. (1998). “The definition and measurement of productivity,” in Melbourne institute of applied economic and social research. Parkville, Victoria.

[B67] Rogers W. A. Kadylak T. Bayles M. A. (2022). Maximizing the benefits of participatory design for human–robot interaction research with older adults. Hum. factors 64 (3), 441–450. 10.1177/00187208211037465 34461761 PMC10645376

[B68] Rozo L. Calinon S. Caldwell D. Jimenez P. Torras C. (2013). Learning collaborative impedance-based robot behaviors. Proc. . AAAI Conf. Artif. Intell. 27 (1), 1422–1428. 10.1609/aaai.v27i1.8543

[B69] Sauer J. Chavaillaz A. Wastell D. (2016). Experience of automation failures in training: effects on trust, automation bias, complacency and performance. Ergonomics 59 (6), 767–780. 10.1080/00140139.2015.1094577 26374396

[B70] Schraft R. D. Meyer C. Parlitz C. Helms E. (2005). PowerMate-A safe and intuitive robot assistant for handling and assembly tasks. IEEE, 4074–4079. 10.1109/robot.2005.1570745

[B71] Sheridan T. B. (2016). Human–robot interaction. Hum. factors 58 (4), 525–532. 10.1177/0018720816644364 27098262

[B72] Shukla A. Srivastava R. (2016). Development of short questionnaire to measure an extended set of role expectation conflict, coworker support and work-life balance: the new job stress scale. Cogent Bus. and Manag. 3 (1), 1. 10.1080/23311975.2015.1134034

[B74] Tian R. Paulos E. (2021). Adroid: augmenting hands-on making with a collaborative robot. New York, NY, USA: ACM, 270.

[B75] Unhelkar V. V. Siu H. C. Shah J. A. (2014). Comparative performance of human and mobile robotic assistants in collaborative fetch-and-deliver tasks. New York, NY, USA: ACM, 82.

[B76] Van Dijk W. Baltrusch S. J. Dessers E. de Looze M. P. (2023). The effect of human autonomy and robot work pace on perceived workload in human-robot collaborative assembly work. Front. Robotics AI 10, 1244656. 10.3389/frobt.2023.1244656 PMC10655125 38023588

[B77] Venkatesh V. Morris M. G. Davis G. B. Davis F. D. (2003). User acceptance of information technology: toward a unified view. MIS Q. 27 (3), 425–478. 10.2307/30036540

[B78] Weidemann C. Mandischer N. van Kerkom F. Corves B. Hüsing M. Kraus T. (2023). Literature review on recent trends and perspectives of collaborative robotics in work 4.0. Robot. (Basel) 12 (3), 84. 10.3390/robotics12030084

[B79] Weiss A. Wortmeier A. Kubicek B. (2021). Cobots in industry 4.0: a roadmap for future practice studies on human-robot collaboration. IEEE Trans. human-machine Syst. 51 (4), 335–345. 10.1109/thms.2021.3092684

[B80] Wolffgramm M. Tijink T. Disberg-Van Geloven M. Corporaal S. (2021). A collaborative robot in the classroom: designing 21st century engineering education together. J. High. Educ. Theory Pract. 21 (16). 10.33423/jhetp.v21i16.4924

[B81] Wongphati M. Osawa H. Imai M. (2015). Gestures for manually controlling a helping hand robot. Int. J. Soc. Robotics 7 (5), 731–742. 10.1007/s12369-015-0302-2

[B82] Zhou E. (2020). The “too-much-of-a-good-thing” effect of job autonomy and its explanation mechanism. Psychol. (Irvine, Calif.) 11 (2), 299–313. 10.4236/psych.2020.112019

