# Supplementary material for: Operators and their human–robot interdependencies: implications of distinct job decision latitudes for sustainable work and high performance
Source: Front Robot AI. 2025 Mar 4;12:1442319. doi: 10.3389/frobt.2025.1442319 (PMC11913812; doi:10.3389/frobt.2025.1442319)
Supplement: Supplementary file 1 [file Supplementaryfile6.docx]

Table A2: Overview of data collection and analysis techniques per sub-concept

| Theory | Data collection | | | Data analysis |
| --- | --- | --- | --- | --- |
| Sub-concept | Data source | Manual work sessions | Collaborative work sessions | Analysis technique |
| Used job decision latitude | Video footage | n.a. | X | Observation template:   - Paused cobot - Reallocated cobot task(s) - Increased cobot speed - Decreased cobot speed - Changed cobot program |
| Need for additional instrumental assistance | Video footage | n.a. | X | Observation template:   - Made request |
| Human-cobot interdependence level | Video footage | n.a. | X | Observation template:   - Cobot tasks - Cobot speed |
| Production reliability | Scorecard | X | X | Submission per keyboard:   - Submitted on time - Assembly error(s) |
| Productivity | Scorecard | X | X | Handling time:   - Mean calculation - Boxplot |
| Motivational characteristics | Work design questionnaire | X | X | Per characteristic:   - Mean calculation - Boxplot |
| Situation awareness | Situation awareness assessments | n.a. | X | Per error level:   - Passed - Failed |
| Automation-induced complacency | Video footage | n.a. | X | Per complacency check:   - Correct handling - Incorrect handling |
| Overall | Voice recording | n.a. | n.a. | Open codes illustrating:   - Job decision latitude - Instrumental assistance - Work perceptions |
