# Supplementary material for: Operators and their human–robot interdependencies: implications of distinct job decision latitudes for sustainable work and high performance
Source: Front Robot AI. 2025 Mar 4;12:1442319. doi: 10.3389/frobt.2025.1442319 (PMC11913812; doi:10.3389/frobt.2025.1442319)
Supplement: Supplementary file 2 [file Supplementaryfile1.docx]

Supplementary Material

Operators and Their Human-Cobot Interdependencies: The Implications of Distinct Job Decision Latitudes for Sustainable Work and High Performance

**Milan Wolffgramm*, Stephan Corporaal, Aard Groen**

*** Correspondence:** Corresponding Author: m.r.wolffgramm@saxion.nl

# Supplementary Data

*Dutch translation of the motivational characteristics section of the Work Design Questionnaire by Morgeson et al. (2006), including response set questions in Dutch and English*

Schaal: Helemaal oneens (1), Oneens (2), Niet oneens / niet eens (3), Eens (4), Helemaal eens (5).

Beantwoord de volgende vragen over de baan waar je ZOJUIST in zat :

Werkplanning autonomie:

- De baan laat me zelf bepalen hoe ik mijn werk inplan
- De baan laat me beslissen in welke volgorde dingen in het werk gedaan worden
- De baan laat me zelf bepalen hoe ik mijn werk doe

Besluitvorming autonomie:

- De baan biedt mij de kans om eigen initiatief of oordeelvorming te gebruiken voor de uitvoer van het werk
- De baan biedt mij de kans om veel beslissingen zelf nemen
- De baan biedt mij genoeg vrijheid om keuzes te maken

Werkmethode autonomie:

- De baan biedt mij de kans zelf te beslissen welke methoden ik gebruik om mijn werk af te maken
- De baan biedt mij genoeg kansen om zelfstandig en vrij te bepalen hoe ik het werk doe
- De baan biedt mij de mogelijkheid zelf te beslissen hoe ik te werk ga

Taakvariatie:

- De baan brengt veel afwisselende taken met zich mee
- De baan bestaat uit het doen van een paar verschillende dingen
- De baan vereist de uitvoering van een breed pakket aan taken
- De baan gaat gepaard met de uitvoer van uiteenlopende taken

Taaksignificatie:

- De resultaten uit mijn werk zullen waarschijnlijk het leven van anderen flink beïnvloeden
- De baan zelf is een erg noemenswaardig en belangrijk onderdeel van een groter geheel
- De baan heeft een grote impact op mensen buiten het bedrijf
- Het uitgevoerde werk heeft een flinke impact op mensen buiten het bedrijf

Taakidentiteit:

- Voor de baan moet een stuk werk met een duidelijk beginpunt en eindpunt gedaan worden
- De baan is zo ingericht dat ik een volledig stuk werk van begin tot eind kan doen
- De baan biedt mij de mogelijkheid om het werk waaraan ik begin helemaal af te maken
- De baan biedt mij de mogelijkheid om werk af te maken waar ik aan begin

Feedback vanuit het werk:

- De taken geven directe en duidelijke informatie over de stand van mijn prestaties
- De baan geeft mij feedback op mijn prestaties
- De baan geeft mij informatie over mijn prestaties

Baancomplexiteit:

- De baan vereist dat ik één taak of activiteit tegelijkertijd doe
- De taken van de baan zijn simpel en niet ingewikkeld
- De baan bevat redelijk makkelijke taken
- *Aan mijn hand zitten zes vingers (response set vraag, geënquêteerd na de werksessie met de cobot)*
  - *Translation: my hand has six fingers (response set question, surveyed after collaborative work session)*
- Voor de baan moeten redelijk simpele taken uitgevoerd worden

Informatieverwerking:

- De baan vereist dat ik veel dingen in de gaten houden
- De baan vereist dat ik veel moet nadenken
- De baan vereist dat ik meerdere dingen tegelijkertijd in de gaten houd
- De baan vereist dat ik een hoop informatie analyseer

Problemen oplossen:

- De baan bevat het oplossen van problemen waar geen kantenklare oplossingen voor zijn
- De baan vereist dat ik creatief ben
- In de baan moet ik vaak omgaan met problemen die nieuw voor mij zijn
- De baan vereist unieke ideeën of oplossingen voor problemen

Variatie in vaardigheden:

- De baan vereist een aantal verschillende vaardigheden
- De baan vereist dat ik een aantal verschillende vaardigheden gebruik om het werk af te kunnen maken
- De baan vereist dat ik een aantal complexe of gevorderde vaardigheden gebruik
- *Ik draag blauwe sokken met gele stippen (response set vraag, geënquêteerd na de manuele werksessie)*
  - *Translation: I wear blue socks with yellow dots (response set question, surveyed after manual work session)*
- De baan vereist dat ik een aantal vaardigheden gebruik

Specialisatie:

- De baan is heel gespecialiseerd als het aankomt op: het doel, de taken of de activiteiten
- De gereedschappen, werkmethoden, materialen enzovoort die gebruikt worden in deze baan zijn alleen voor dit werk te gebruiken
- De baan vereist hele specifieke kennis en vaardigheden
- De baan vereist diepgaande kennis en expertise
