# Supplementary material for: Operators and their human–robot interdependencies: implications of distinct job decision latitudes for sustainable work and high performance
Source: Front Robot AI. 2025 Mar 4;12:1442319. doi: 10.3389/frobt.2025.1442319 (PMC11913812; doi:10.3389/frobt.2025.1442319)
Supplement: Supplementary file 3 [file Supplementaryfile5.docx]

Table A1: Motivational characteristics under study, derived from Morgeson and Humphrey (2006)

| **Motivation Characteristic** | **Definition** |
| --- | --- |
| Work scheduling autonomy | The extent to which a job allows the operator freedom, independence, and discretion to schedule work (Morgeson et al., 2006). |
| Work methods autonomy | The extent to which a job allows the operator freedom, independence, and discretion to choose the methods used to perform tasks (Morgeson et al., 2006). |
| Decision-making autonomy | The extent to which a job allows the operator freedom, independence, and discretion to make decisions (Morgeson et al., 2006). |
| Skills variety | The extent to which a job requires the operator to use a variety of different skills to complete the work (Hackman, 1980). |
| Task variety | The degree to which a job requires the operator to perform a wide range of tasks on the job (Morgeson et al., 2006). |
| Significance | Reflects the degree to which the operator’s job influences the lives or work of internal or external others (Hackman and Oldham, 1975) |
| Task identity | The degree to which the operator’s job involves a whole piece of work, the results of which can be easily identified (Sims, Szilagyi, and Keller, 1976). |
| Feedback from job | The degree to which the operator’s job provides direct and clear information about the effectiveness of task performance (Hackman and Oldham, 1976). |
| Job complexity | To the extent to which the tasks on the operator’s job are complex and difficult to perform (Campion, 1988). |
| Specialisation | The extent to which the operator’s job involves performing specialised tasks or possessing specialised knowledge and skills (Campion, 1988). |
| Information processing | Reflects the degree to which a job requires attending to and processing data or other information (Morgeson et al., 2006). |
| Problem-solving | The degree to which the operator’s job requires unique ideas or solutions and reflects the more active cognitive processing requirements of a job (Jackson et al., 1993). |

**References**

Campion, M. (1988). Interdisciplinary approaches to job design: a constructive replication with extensions. *J. Appl. Psychol. 73* (88), 467–481. doi:10.1037//0021-9010.73.3.467

Hackman, J. R. (1980). Work redesign and motivation. *Prof. Psychol. 11* (3), 445–455. doi:10.1037/0735-7028.11.3.445

Hackman, J. R., and Oldham, G. R. (1975). Development of the job diagnostic survey. *J. Appl. Psychol. 60* (2), 159–170. doi:10.1037/h0076546

Hackman, J. R., and Oldham, G. R. (1976). Motivation through the design of work: test of a theory. *Organ. Behav. Hum. Perform. 16* (2), 250–279. doi:10.1016/0030-5073(76)90016-7

Jackson, P. R., Wall, T. D., Martin, R., and Davids, K. (1993). New measures of job control, cognitive demand, and production responsibility. *J. Appl. Psychol. 78* (5), 753–762. doi:10.1037//0021-9010.78.5.753

Morgeson, F. P., and Humphrey, S. E. (2006). The Work Design Questionnaire (WDQ): developing and validating a comprehensive measure for assessing job design and the nature of work*. J. Appl. Psychol. 91* (6), 1321–1339. doi:10.1037/0021-9010.91.6.1321

Sims, H. P., Szilagyi, A. D., and Keller, R. T. (1976). The measurement of job characteristics. *Acad. Manag. J. 19* (2), 195–212. doi:10.5465/255772
