# Supplementary material for: Operators and their human–robot interdependencies: implications of distinct job decision latitudes for sustainable work and high performance
Source: Front Robot AI. 2025 Mar 4;12:1442319. doi: 10.3389/frobt.2025.1442319 (PMC11913812; doi:10.3389/frobt.2025.1442319)
Supplement: Supplementary file 4 [file Supplementaryfile3.docx]

Supplementary Material

Operators and Their Human-Cobot Interdependencies: The Implications of Distinct Job Decision Latitudes for Sustainable Work and High Performance

**Milan Wolffgramm*, Stephan Corporaal, Aard Groen**

*** Correspondence:** Corresponding Author: m.r.wolffgramm@saxion.nl

# Supplementary Data

*Dutch debriefing questions:*

1. Wat vond je in deze werksessie met de cobot het meeste meevallen?
2. Wat vond je in deze werksessie met de cobot het meest tegenvallen?
3. Wat vond je van het werk dat je gedaan hebt?
4. Wat vond je van de hulp die je kreeg tijdens de werksessie?
5. Wat vond je van de keuzevrijheid die je had tijdens de werksessie?

*English translation:*

1. What did you like most about this work session with the cobot?
2. What did you dislike most about this work session with the cobot?
3. What do you think about the tasks you performed during this simulation?
4. What do you think of the assistance that was provided to you during the simulation?
5. What do you think about the decision-making freedom you had during the simulation?
