# Supplementary material for: Operators and their human–robot interdependencies: implications of distinct job decision latitudes for sustainable work and high performance
Source: Front Robot AI. 2025 Mar 4;12:1442319. doi: 10.3389/frobt.2025.1442319 (PMC11913812; doi:10.3389/frobt.2025.1442319)
Supplement: Supplementary file 5 [file Supplementaryfile2.docx]

Supplementary Material

Operators and Their Human-Cobot Interdependencies: The Implications of Distinct Job Decision Latitudes for Sustainable Work and High Performance

**Milan Wolffgramm*, Stephan Corporaal, Aard Groen**

*** Correspondence:** Corresponding Author: m.r.wolffgramm@saxion.nl

# Supplementary Data

*Dutch probes for situation awareness error-level 1 (SA1), error level 2 (SA2), and error level 3 (SA3):*

1. Noem drie dingen op het witte blad voor je. (SA1)
2. Noem drie taken die de cobot kan uitvoeren. (SA2)
3. Wat stond er op de toets die je als laatst in je toetsenbord drukte? (SA2)
4. Wat gaat de cobot doen als je op ‘Start’ zou drukken? (SA3)
5. Welke kleur had de cobot zijn LED-ring voordat de cobot stopte? (SA1)
6. Hoeveel toetsenborden denk je in dit tempo nog af te kunnen maken voordat de tijd om is? (SA3)
7. Hoe kan je waarnemen dat de cobot aan het bewegen is? (SA1)
8. Wat doet de cobot als de LED-ring een regenboog kleur laat zien? (SA2)
9. Hoeveel toetsen moet je nog in je toetsenbord prikken voordat die af is? (SA3)

English translation:

1. Name three things presented on the white sheet in front of you. (SA1)
2. Name three tasks the cobot can perform. (SA2)
3. What was on the last key you inserted in your keyboard? (SA2)
4. What will the cobot do once you press 'Play'? (SA3)
5. What color was on the cobot's LED ring before the cobot stopped moving? (SA1)
6. With your current working pace, how many keyboards do you think you will complete before time's up? (SA3)
7. How can you sense the cobot's movement? (SA1)
8. What is the cobot doing if it shows rainbow-colored LED lights? (SA2)
9. How many keys must you insert in your keyboard before it's complete? (SA3)
