# Supplementary material for: Operators and their human–robot interdependencies: implications of distinct job decision latitudes for sustainable work and high performance
Source: Front Robot AI. 2025 Mar 4;12:1442319. doi: 10.3389/frobt.2025.1442319 (PMC11913812; doi:10.3389/frobt.2025.1442319)
Supplement: Supplementary file 6 [file Supplementaryfile4.docx]

Supplementary Material

Operators and Their Human-Cobot Interdependencies: The Implications of Distinct Job Decision Latitudes for Sustainable Work and High Performance

**Milan Wolffgramm*, Stephan Corporaal, Aard Groen**

*** Correspondence:** Corresponding Author: m.r.wolffgramm@saxion.nl

# Supplementary Data


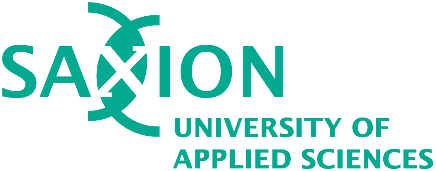


Protocol Human-Cobot Observation Study

Author: Milan Wolffgramm

Property: Research Group Employability Transition, Saxion University of Applied Sciences

Version: 8

Translated: October 28th, 2023

1. Important Information for Researchers

1. Stick to the protocol throughout the simulation and do NOT diverge from the protocol.
2. Already fill out the participant number on the tablet.
3. Discuss the goal of the simulation with Operator once the simulation has been completed.
4. Do not mention what will come during the simulation (even if Operator asks for this information); only communicate the information presented in this protocol.
5. Keep in mind that Operator might consider things difficult you yourself find obvious, act without judgment.
6. Determine who will fulfil the **Foreman** role and who will fulfil the **Warehouse Worker** role and help each other throughout the simulation
7. Help Operator when your assistance is required and help to the best of your abilities.
8. You are responsible for Operator’s well-being and safety as long as the simulation runs.
   - Keep a one-and-a-half meter distance from Operator and wear a facemask.
   - Be extra alert when the cobot is moving and intervene if necessary.
9. Make sure that the keyboards and grids are being resupplied to prevent your simulation from freezing when running.
10. Be precise regarding the resupply of keyboards and grids. Flawed resupply has consequences for your simulation and, thus, the outcomes.
11. Leave the lab room properly: ready for future usage (charged tablets, full grids, organised tables, cobot in home position).

2. COVID-19 Measures

**Note for researchers:**

- Hand sanitising gel, facemasks, and gloves are available.
- Keep one-and-a-half meter distance from Operator, fellow researchers, and others.
- The maximum amount of persons in the lab space should not be exceeded. Keep an eye on this.
- Check if Operator brough a facemask. If not, offer one.
- Assure Operator disinfects its hands before entering.
- It is not mandatory for Operator to wear gloves. It is allowed though.
- It is not mandatory for Operator to wear a facemask when sitting at the table.
- Researchers must wear a facemask if they cannot maintain a one-and-a-half meter distance.
- Researchers are advised to wear a facemask as much as possible.
- It is not mandatory for researchers to wear gloves. It is allowed though.
- Operator must be advised to avoid contact with vulnerable individuals for the upcoming ten days.

**Task Foreman**: communicate the following to Operator:

- “Did you bring a facemask?”
  - If yes: continue protocol.
  - If no: offer facemask and continue protocol.
- “Could you please disinfect your hands?”
- “Would you like to wear gloves? This is not mandatory.”
  - If yes: offer glove box and continue protocol.
  - If no: continue protocol.
- “When you are seated at the table, you are not obligated to wear a facemask.”
- “I will wear a facemask if I cannot keep a one-and-a-half-meter distance.”
- “We did everything we could to run this simulation safely. Nonetheless, it is recommended to avoid contact with vulnerable individuals for the upcoming ten days.”

3. General Explanation About the Simulation

**Task Foreman**: communicate the following to Operator:

- “Again, welcome. I will start by explaining to you what is intended.”
- “Make yourself comfortable, adjust your seat if necessary.”
- “You are an operator working for an international manufacturer of customised keyboards.”
- “I am your Foreman; over there is Warehouse Worker who prepares the working activities.”
- “Different orders were placed by our customers and you will be processing these orders.”
- “We will conduct two 45-minute work sessions; a small break follows after completing the first work session.”
- “Eating, drinking, toilet breaks, and phone usage are prohibited during the work sessions. Of course, these activities are allowed during the break. Warm drinks and water could be acquired during the break.
- “A logbook was created for you to fill out something or give your opinion about the tasks you are performing in this space. I will let you know when to get out logbook.”
- “Furthermore, it is important to inform you that you can stop the work sessions at any time you want.”
- “You are also entitled to a summary of this study when available.”
- “Do you have any questions at the moment?”
  - If yes: answer question(s).
  - If no: “could you please get out your logbook and read and fill out the consent form? (Appendix A)”

4. Manual Work Session

4.1 General Explanation by Foreman

**Task Foreman**: communicate the following to Operator:

- “This is your workstation for the first work session.”
- “You will be assembling keyboards.”
- “We work with different keyboard brands and each keyboard must be equipped with 40 keys.”
- “Which keyboard brands to assemble, how many keyboards to assemble, and where to insert the keys is being explained in the order booklet (Appendix B). That is the white booklet lying next to you. We will go through the booklet in a minute.”
- “Your goal is to assemble the keyboards correctly at a working pace you could keep up an entire working day. Do not work too hard, but imagine that you have to conduct the working activities for a long time. You mustn't take longer than seven-and-a-half minutes to assemble a keyboard and submit it by placing it on the plastic plate.”
- “It is important to mention that one incorrect key insertion, for instance, because it is inserted at the wrong place or because it is oriented incorrectly, will result in a product defect. The defective product will be returned to you for repair.”
- “Before I show you what to do through a step-by-step demonstration experience, is the overall idea clear to you?”
  - If yes: continue to demo.
  - If no: answer question(s).

4.2 Manual Demonstration Guided by Foreman, Executed by Operator:

**Task Foreman:** communicate the following to Operator:

- “How are you conducting your working activities:
  1. Warehouse Worker will supply you with the keyboard and grid with keys in sockets.
  2. You open the booklet.
  3. You check the order. You will see information explaining the keyboard brand, the number of keyboards to assemble, the order number and associated barcode, which keys you need, and where to insert these keys into the keyboard.
  4. You should check the keyboard’s barcode on the back to verify that it matches the barcode in the booklet.
  5. You look for the right keys in the grid.
  6. You remove the keys from their sockets. Please leave the sockets in the grid or back them on the green lines.
  7. You check whether the key orientation is upside down or sideward.
  8. You insert the key gently into the keyboard. Do not use too much force. If you are unable to insert a key, placing it on top of the designated spot suffices.
  9. You may be provided with keys you do not need. You can leave these keys on the grid.
  10. Finally, check if all keys are inserted at the right spot and oriented correctly.
  11. Submit the keyboard by placing it on the plastic plate.
  12. You will proceed to the order’s next keyboard or, if all keyboards have been submitted, you proceed to the next order.”
- “If you have questions during the work session, feel free to ask me or Warehouse Worker. You are allowed to ask us anything.”
- “In case you want to make notes, you can use the notebook and pencil next to you.”
- “If you cannot get a key out of its socket or your keyboard, you can carefully try to get it out using your nails or the tiny screwdriver.”
- “There is a time available showing you the work session’s remaining time. This work session’s duration is 45 minutes.”
- “Would you like me to turn on the radio during the work session?”
- “Do you have any questions?”
  - If yes: answer question(s).
  - If no: start work session.

**Task Warehouse Worker**: position keyboards and grid for the first order.

**Task Warehouse Worker**: turn on the camera and ensure it is well-positioned towards Operator.

4.3 During the Work Session:

**Task Foreman**: turn on the radio if desired by Operator.

**Task Warehouse Worker and Foreman**: replace the grid when the keyboard has been submitted.

**Task Warehouse Worker and Foreman**: fill the holder with new keyboards as soon as the order’s last keyboard has been submitted.

**Task Foreman**: read the time on the timer when a keyboard has been placed on the plastic plate.

**Task Foreman**: verify if the keyboard has been assembled according to the assembly instructions. Return the keyboard back once to Operator if at least one key has been assembled incorrectly. The keyboard must be repaired and resubmitted before other working activities can be resumed.

**Task Foreman**: fill out the scorecard (Appendix C).

**Task Foreman**: hand over the assembled keyboard and grid to Warehouse Worker.

**Task Warehouse Worker**: disassemble the keyboard and fill the grid using the grid-filling instructions (Appendix D).

4.4 Finalising Manual Work Session by Foreman
**Task Foreman:** communicate the following to Operator:

- “Alright, time is up. Please take out your logbook and fill out the questionnaire about the tasks you performed for the last 45 minutes (Appendix E). Again, it is important to fill you the questionnaire promptly. After this, you have a fifteen-minute break.”
- “Thank you for filling out the questionnaire. You are on a fifteen-minute break now.”

**Task Foreman**: write the manual work session’s productivity and production reliability outcomes on separate sticky notes.

**Task Warehouse Worker**: turn off the camera.
 **Task Warehouse Worker**: sort the keyboards and grids based on the order sequence.

**Task Warehouse Worker**: add sockets with A, B, and C keys at the grids used for the second order.

**Task Warehouse Worker:** place the order booklet, notebook, pencil, and screwdriver at the second workstation.
 **Task Warehouse Worker**: check if the cobot’s speed is at 75%. If not, adjust cobot speed to 75%.

5. Collaborative Work Session

5.1 General Explanation by Foreman
**Task Warehouse Worker**: turn on the camera and ensure it is well-positioned towards Operator and cobot.

**Task Foreman:** communicate the following to Operator:

- “This is your workstation for the second work sessions.”
- “You will assemble keyboards again. The assembly process is exactly the same as the processes of the manual work session. All the steps are identical, and you will work on the same orders.”
- “This time, you will assemble the keyboards together with the cobot. In a moment, I will show you step by step how to do this.”
- “It is still important to correctly assemble the keyboards at a maintainable pace and within seven-and-a-half minutes per keyboard. If possible, use the cobot to improve your manual performance and make your work more maintainable. We will provide you with the grids and keyboards.”
- “Before we proceed, is the general overall idea clear to you?”
  - If yes: proceed to safety instructions.
  - If no: answer question(s).

5.2 Safety Instructions by Foreman

**Task Foreman**: communicate the following to Operator:

- “We start with a part on safety. You will share your workplace with a fast and strong device. The cobot is unable to see you but stops when colliding with you. Colliding with the cobot is strongly prohibited.”
- “Be extra alert when moving your hands into the white wild. We intentionally projected images and a safety banner. This is because the cobot operates in this area and places its sockets in there. The cobot could collide with you or place sockets on your hand if you do not pay enough attention.”
- “In other words, be extra alert when your hands are in the white field. You are not allowed to pick sockets from the white field while looking into the order booklet.”

**Task Foreman**: point towards projected images in white field (Appendix F).

**Task Foreman**: communicate the following to Operator:

- “Pay attention when the cobot is moving. You can hear the cobot moving and you will see the LED-ring light up.”
- “If you want the cobot to stop moving, you can press the ‘stop’ button on the left bottom of the screen.”
- “The red button is the emergency stop. You are allowed to press the emergency stop at any time. I also have an emergency stop at my side of the workstation.”
- “Before we proceed, do you have questions about your safety?”
  - If yes: answer question(s) and proceed to demonstration.
  - If no: proceed to demonstration.

5.3 General Cobot Demonstration Guided by Foreman, Executed by Operator:

**Task Foreman**: check if the cobot’s speed is at 75%. If not, adjust cobot speed to 75%.
 **Task Foreman**: communicate the following to Operator:

- “A cobot program has been created for each keyboard brand. The program names correspond with the keyboard names in the order booklet.”

**Task Foreman**: ask Operator to open the Demo program.

**Task Foreman**: communicate the following to Operator:

- “Click on the right program and then click on ‘Open’.”
- “The build-up of all programs is for each keyboard brand the same. The cobot is capable of doing four things. We will go through each of these step by step:
  1. The cobot can scan the keyboard’s barcode and tell you whether the barcode corresponds with the cobot program.
  2. The cobot is capable of picking up the keyboard and handing it to you.
  3. The cobot is capable of picking the sockets and placing these in order from left to right and from top row to bottom row.
  4. The cobot is capable of doing a final inspection and letting you know whether you assembled the keyboard correctly or incorrectly.”

**Task Foreman**: ask Operator to activate the ‘Scan Barcode’ task.

**Task Foreman**: communicate the following to Operator:

- “Proceed as follows:
  1. Choose which of the four tasks you would like to activate.
  2. Double click on the grey row starting the triangle icon and then click ‘Unsuppress’. The task will become active.
  3. Centre the cobot by clicking the ‘Auto’ button. Hold the button clicked until you see a green check mark in the bottom right corner of the screen.
  4. When you press ‘Play’, the cobot will execute the task. Remember your safety.
  5. Once the cobot has executed its task, it will fall into a standstill and turn off.
  6. You will have to lock the task by going to the row with the triangle icon—this is the row under the task name.
  7. Next you click on the tab ‘Structure’.
  8. In the right bottom corner, you will see a ‘Suppress’ button. The entirety of task rules should be compressed, and the row with the triangle should be greyed out.
  9. Now you can unsuppress another task, play it, and suppress it.

**Task Foreman**: ask Operator to unsupress, play, and suppress the ‘Pick Board’ task.

**Task Foreman**: ask Operator to unsupress, play, and suppress the ‘Pick Keys’ task.

**Task Foreman**: communicate the following to Operator:

- “In case you want to get rid of the sockets, you can slide these into the ditch.”
- “Make sure the cobot has enough space to place the sockets on the table. If not, the cobot will go into a safety stop.”

**Task Foreman**: ask Operator to unsupress, play, and suppress the ‘Scan Board’ task.

**Task Foreman**: communicate the following to Operator:

- “If you want the cobot to scan your keyboard. Put it in the zone with the blue border in front of you.”

5.4 Ascribing a Scenario

**Task Warehouse Worker**: provide the bin with folded scenario cards.

**Task Foreman**: communicate the following to Operator:

- “Could you please pick a card from the bin and tell me which number is on the card. After that, you can throw away the card.”

**Task Warehouse Worker**: remove the bin with scenario cards once the operator picked a card and make sure Operator did not put its scenario card back in the bin.

**Task Foreman**: based on the Operator’s scenario card, proceed to the instructions for Scenario 1 (5.4.1), Scenario 2 (5.4.2) or Scenario 3 (5.4.3).

5.4.1 Information Scenario 1 and Finishing Demonstration

**Task Foreman**: communicate the following to Operator:

- “In our company, we find it important that that the cobot is being used.”
- “Therefore, all cobot tasks must be used.”
- “Furthermore, you have to run the cobot tasks chronologically. In other words, first scanning the barcode, then picking the keyboard, then collecting the keys, and then running the final check.”
- “Moreover, the cobot’s speed should remain at 75%.”
- “However, you are allowed to temporarily freeze the cobot’s movement by pressing the ‘Pause’ button in the bottom of the screen. Press ‘Play’ to unfreeze the cobot. Make sure to press the ‘Pause’ button and not the ‘Stop’ button next to it if you only want to temporarily freeze the cobot. When pressing the ‘Stop’ button, the cobot will require re-centering and start its task again.”
- “Finally, the build-up of the cobot’s programs cannot be modified.”
- “If you have any questions during the work session, feel free to ask me or Warehouse Worker.”

**Task Foreman**: communicate the following to Operator:

- “Now we will close the Demo program and open the first order’s program.”
- “If you want to open another program, you take the following steps:
  1. Go to the word File with your cursor in the upper left corner of the screen.
  2. Click on ‘Open program’.
  3. Click on ‘Discard changes’.
  4. Next, you will find yourself an overview of all cobot programs.
  5. Click on the program you would like to open.
  6. Click on ‘Open’ for the program to open.

5.4.2 Information Scenario 2 and Finishing Demonstration
**Task Foreman**: communicate the following to Operator:

- “In our company, we find it important that that the cobot is being used.”
- “Therefore, we encourage you to use the cobot actions that you think suite you. You can choose which cobot tasks to use.”
- “Furthermore, you are encouraged to consider adjusting the cobot speed. You can adjust the cobot speed by using the switch in the bottom center of the screen. If you click on the lever and drag it to the left, the cobot speed will decrease. Dragging the lever to the right will increase the cobot speed.”
- “You are allowed to temporarily freeze the cobot’s movement by pressing the ‘Pause’ button in the bottom of the screen. Press ‘Play’ to unfreeze the cobot. Make sure to press the ‘Pause’ button and not the ‘Stop’ button next to it if you only want to freeze the cobot. The ‘Stop’ button, requires re-centering and the cobot will start the task from the start.”
- “However, the build-up of the cobot’s programs cannot be modified.”
- “Moreover, the cobot’s speed should remain at 75%.”
- “Finally, the cobot’s programs cannot be modified.”
- “If you have any questions during the work session, feel free to ask me or Warehouse Worker.”

**Task Foreman**: communicate the following to Operator:

- “Now we will close the Demo program and open the first order’s program.”
- “If you want to open another program, you take the following steps:

1. Go to the word File with your cursor in the upper left corner of the screen.
2. Click on ‘Open program’.
3. Click on ‘Discard changes’.
4. Next, you will find yourself an overview of all cobot programs.
5. Click on the program you would like to open.
6. Click on ‘Open’ for the program to open.

5.4.3 Information Scenario 3 and Finishing Demonstration

**Task Foreman**: communicate the following to Operator:

- “In our company, we find it important that that the cobot is being used.”
- “Therefore, we encourage you to use the cobot actions that you think suite you. You can choose which cobot tasks to use.”
- “Furthermore, you are encouraged to consider adjusting the cobot speed. You can adjust the cobot speed by using the switch in the bottom center of the screen. If you click on the lever and drag it to the left, the cobot speed will decrease. Dragging the lever to the right will increase the cobot speed.”
- “You are allowed to temporarily freeze the cobot’s movement by pressing the ‘Pause’ button in the bottom of the screen. Press ‘Play’ to unfreeze the cobot. Make sure to press the ‘Pause’ button and not the ‘Stop’ button next to it if you only want to temporarily freeze the cobot. When pressing the ‘Stop’ button, the cobot will require re-centering and start its task again.”
- “Moreover, the cobot’s speed should remain at 75%.”
- “Moreover, you are encouraged to change the build-up of at least one cobot program by yourself or have the build-up changed by us. For instance, when there are rules in the program you do not like and want to be suppressed or in case you have other ideas to better align the build-up of the programs with your needs. You do not have to do this yourself if you do not want to. Simply tell us what you would like to have adjusted. There are no wrong ideas. We will check on the spot if and how these adjustments can be arranged.”
- “If you have any questions during the work session, feel free to ask me or Warehouse Worker.”

**Task Foreman**: communicate the following to Operator:

- “Now we will close the Demo program and open the first order’s program.”
- “If you want to open another program, you take the following steps:

1. Go to the word File with your cursor in the upper left corner of the screen.
2. Click on ‘Open program’.
3. Click on ‘Discard changes’.
4. Next, you will find yourself an overview of all cobot programs.
5. Click on the program you would like to open.
6. Click on ‘Open’ for the program to open.

5.4 Final Announcements by Foreman

**Task Foreman**: communicate the following to Operator:

- “To summarize, assemble to the keyboards without any defect and within seven-and-a-half minutes per keyboard.”
- “Use the cobot in the way we just discussed use to improve your manual performance and make your work more maintainable.”
- “Do you have any questions?”
  - If yes: answer question(s).
  - If no: “please take out your logbook and fill out the questionnaires about the work session you are about to start and you opinion about the cobot. Fill out the questionnaires promptly.”

**Task Warehouse Worker**: position keyboards and grid for the first order.

**Task Foreman**: insert scenario number in the scorecard.

5.5 During the Work Session:

**Task Warehouse Worker and Foreman**: replace the grid when the keyboard has been submitted.

**Task Warehouse Worker and Foreman**: fill the holder with new keyboards as soon as the order’s last keyboard has been submitted.

**Task Foreman**: pay constant attention to Operator’s safety.

**Task Foreman**: check the monitors to see if the keyboard matches the order and whether the keyboards are assembled correctly. Give a signal using the remote control. Be aware of the following: the LED-ring can only be remotely controlled when showing a rainbow pattern.

**Task Foreman**: read the time on the timer when a keyboard has been placed on the plastic plate.

**Task Foreman**: verify if the keyboard has been assembled according to the order’s assembly drawing. The keyboard must be repaired and resubmitted before other working activities can be resumed.

**Task Foreman**: fill out the scorecard.

**Task Foreman**: hand over the assembled keyboard and grid to Warehouse Worker.

**Task** **Warehouse Worker**: disassemble the keyboard and fill the grid using the grid-filling drawing.

5.5.1 Around 30 Minutes Remaining on Timer: Foreman Conducts Alertness Check 1
**Task Foreman**: take out the alertness sheet (Appendix G).

**Task Foreman**: determine the right answers for the situation awareness questions 3 and 4.

**Task Foreman**: pause the time around the 30-minute mark.

**Task Foreman**: cover the timer and freeze the cobot using the remote control.

**Task Foreman**: communicate the following to Operator:

- “We briefly pause the work session. I paused the timer and the cobot. Do no longer look at your workstation, and turn your chair away if necessary. Answer the following questions out loud:

1. Name three things presented on the white sheet in front of you.
2. Name three tasks the cobot can perform.
3. What was on the last key you inserted in your keyboard?
4. What will the cobot do once you press ‘Play’?”

**Task Foreman**: note down Operator’s answers.

**Task Foreman**: communicate the following to Operator:

- “Thank you, please proceed.”

**Task Foreman:** continue timer.

**Task Foreman:** determine whether Operator’s answers are correct and fill out the alertness sheet.

5.5.2 Around 20 Minutes Remaining on Timer: Foreman Conducts Alertness Check 2
**Task Foreman**: take out the alertness sheet.

**Task Foreman**: determine the right answers for the situation awareness questions 5 and 7.

**Task Foreman**: pause the time around the 20 minute mark.

**Task Foreman**: cover the timer and freeze the cobot using the remote control.

**Task Foreman**: communicate the following to Operator:

- “We briefly pause the work session again. I paused the timer and the cobot. Do no longer look at your workstation and turn your chair away if necessary. Answer the following questions out loud:

1. What colour was on the cobot’s LED ring before the cobot stopped moving?
2. With your current working pace, how many more keyboards do you think to complete before time’s up?”

**Task Foreman**: note down Operator’s answers

.

**Task Foreman**: communicate the following to Operator:

- “Thank you, please proceed.”

**Task Foreman:** continue timer.

**Task Foreman:** determine whether Operator’s answers are correct and fill out the alertness sheet.

5.5.3 Around 10 Minutes Remaining on Timer: Foreman Conducts Alertness Check 3

**Task Foreman**: take out the alertness sheet.

**Task Foreman**: determine the right answers for the situation awareness question 10.

**Task Foreman**: pause the time around the 20 minute mark.

**Task Foreman**: cover the timer and freeze the cobot using the remote control.

**Task Foreman**: communicate the following to Operator:

- “We briefly pause the work session again. I paused the timer and the cobot. Do no longer look at your workstation and turn your chair away if necessary. Answer the following questions out loud:

1. How can you sense the cobot’s movement?
2. What is the cobot doing if it shows rainbow-coloured LED lights?
3. How many keys must you insert in your keyboard before it's complete?”

**Task Foreman**: note down Operator’s answers.

**Task Foreman**: communicate the following to Operator:

- “Thank you, please proceed.”

**Task Foreman:** continue timer.

**Task Foreman:** determine whether Operator’s answers are correct and fill out the alertness sheet.

5.6 Finalising Collaborative Work Session by Foreman

**Task Foreman**: communicate the following to Operator:

- “Alright, time is up. Please take out your logbook and fill out the last questionnaire about the tasks you just performed for the last 45 minutes. Again, it is important to fill you the questionnaire promptly.”
- “As soon as you are done, we will shortly debrief and finalize the simulation.”

**Task Foreman**: write the manual work session’s productivity and production reliability outcomes on separate sticky notes.

**Task Foreman**: gather the sticky notes from both work sessions and bring these to the debriefing interview.

5.7 Debriefing Interview

**Task Foreman**: ask Operator the following questions and ask a follow-up question if necessary:

1. What did you like most about this work session with the cobot?
2. What did you dislike most about this work session with the cobot?
3. What do you think about the tasks you performed during this simulation?
4. What do you think of the assistance that was provided to you during the simulation?
5. What do you think about the decision-making opportunities you had during the simulation?

**Task Foreman**: put the manual and collaborative sticky notes on the white board and ask for Operator’s reflection on the differences. Raise a follow-up question if deemed necessary.

**Task Foreman**: communicate the following to Operator:

- “Do you have any remaining questions or matters you would like to discuss?”
  - If yes: respond to Operator’s input.
  - If no: conclude the simulation.

**Task Warehouse Worker**: turn off the camera.

**Task Warehouse Worker**: remove sockets with A, B, and C keys from the grids used for the second order.

**Task Warehouse Worker**: sort the keyboards and grids based on the order sequence.

**Task Warehouse Worker:** place the order booklet, notebook, pencil, and screwdriver at the first workstation.

**Task Warehouse Worker**: check if the cobot’s speed is 75%. If not, adjust cobot speed to 75%.

5.8 Finalising Simulation by Foreman
**Task Foreman**: provide Operator with participation incentive
 **Task Foreman**: inform Operator that it is possible to contact the research team in case of questions or any other matters related to participating in the simulation.

*End of protocol*

Appendices A-G

Available upon request.
